# The role of vegetative cell fusions in the development and asexual reproduction of the wheat fungal pathogen *Zymoseptoria tritici*

**DOI:** 10.1186/s12915-020-00838-9

**Published:** 2020-08-11

**Authors:** Carolina Sardinha Francisco, Maria Manuela Zwyssig, Javier Palma-Guerrero

**Affiliations:** 1 Plant Pathology Group, Institute of Integrative Biology, ETH Zürich, 8092 Zürich, Switzerland; 2 New Address: Department of Biointeractions and Crop Protection, Rothamsted Research, Harpenden, UK

**Keywords:** Cell-to-cell communication, Anastomosis, Vegetative growth, Melanization, Asexual reproduction

## Abstract

**Background:**

The ability of fungal cells to undergo cell-to-cell communication and anastomosis, the process of vegetative hyphal fusion, allows them to maximize their overall fitness. Previous studies in a number of fungal species have identified the requirement of several signaling pathways for anastomosis, including the so far best characterized *soft* (*So*) gene, and the MAPK pathway components MAK-1 and MAK-2 of *Neurospora crassa*. Despite the observations of hyphal fusions’ involvement in pathogenicity and host adhesion, the connection between cell fusion and fungal lifestyles is still unclear. Here, we address the role of anastomosis in fungal development and asexual reproduction in *Zymoseptoria tritici*, the most important fungal pathogen of wheat in Europe.

**Results:**

We show that *Z. tritici* undergoes self-fusion between distinct cellular structures, and its mechanism is dependent on the initial cell density. Contrary to other fungi, cell fusion in *Z. tritici* only resulted in cytoplasmic mixing but not in multinucleated cell formation. The deletion of the *So* orthologous *ZtSof1* disrupted cell-to-cell communication affecting both hyphal and germling fusion. We show that *Z. tritici* mutants for MAPK-encoding *ZtSlt2* (orthologous to *MAK-1*) and *ZtFus3* (orthologous to *MAK-2*) genes also failed to undergo anastomosis, demonstrating the functional conservation of this signaling mechanism across species. Additionally, the *ΔZtSof1* mutant was severely impaired in melanization, suggesting that the *So* gene function is related to melanization. Finally, we demonstrated that anastomosis is dispensable for pathogenicity, but essential for the pycnidium development, and its absence abolishes the asexual reproduction of *Z. tritici*.

**Conclusions:**

We demonstrate the role for *ZtSof1*, *ZtSlt2*, and *ZtFus3* in cell fusions of *Z. tritici*. Cell fusions are essential for different aspects of the *Z. tritici* biology, and the *ZtSof1* gene is a potential target to control septoria tritici blotch (STB) disease.

## Background

Communication is a ubiquitous primitive characteristic developed by all living species. The ability to communicate effectively may affect mating, predation, competition, dominance hierarchy, signal modalities, and survival [1–3]. This complex mechanism starts when a given organism (the sender) secretes in the environment a self-produced molecular signal (the message) that alters the behavior of another organism (the receiver) [1, 3]. Communication also happens at the cellular level. This so-called cell-to-cell communication creates a complex signaling network that involves different extracellular signals and distinct cell types that regulate several pathways [4–6]. Inter- and intraspecies cell-to-cell communication has been widely studied in fungi to address biological functions including the secretion of pheromones to attract the opposite sexual partner, the production of quorum sensing molecules controlling the expression of virulence factors or morphological changes, and the regulation of cell fusions during vegetative growth [6–9].

The fungal mycelium is formed by three integrated processes, including hyphal extension, branching, and vegetative hyphal fusion (VHF) (also known as anastomosis) [10]. In this last-mentioned process, two growing cells with identical vegetative compatibility loci engage in cell-to-cell communication, which is thought to involve the secretion of unknown diffusible molecules and results in re-direction of polarized hyphal growth toward each other. After physical contact, the cell walls are remodeled, the plasma membranes fuse, and the two interconnected cells exchange cytoplasm and organelles [11]. When the anastomosed individuals are vegetatively incompatible, the two fused cells rapidly collapse following DNA degradation by programmed cell death, or they are severely inhibited in their growth [12]. It is widely accepted that mycelial network formed through VHF facilitates the intra-hyphal communication, translocation of water and nutrients, and signal molecules, which improve the general homeostasis and spatial expansion of the fungal colony [13, 14]. In some fungi, hyphal fusion is required for pathogenicity and host adhesion [15–17]. Cell fusions can also occur between germinating conidia, the asexual spores of many fungi. The process of fusion between germinating conidia involves the formation and interaction of specialized hyphae, so-called conidial anastomosis tubes (CATs). CATs are thinner and shorter than vegetative hyphal fusions (VHFs), and its induction is dependent on nutrient deprivation and initial cell density [18]. Cell fusions may serve to improve colony establishment, as well as to increase the genetic variability by facilitating heterokaryosis and parasexual recombination [18]. Gene or chromosome transfers by cell fusions between individuals of the same or different species allow certain fungi to acquire pathogenicity or to broaden their host specificity [19–21]. Albeit non-self-anastomoses are described [22, 23], this might be a rare event in nature.

In the last decades, different studies about the molecular mechanisms underlying cell fusion identified several mutants defective in anastomosis, revealing that fungal communication and fusion are complex mechanisms that encompass several signaling pathways [6]. The best-characterized mutant is the *soft* (*So*) gene of *Neurospora crassa* [24]. So, it is proposed to be the scaffold protein for some mitogen-activated protein kinase (MAPK) from the cell wall integrity (CWI) signaling pathway implicated in the regulation of different fungal processes [25–27]. For instance, *So* contributes to septal plugging during hyphal injury or damage caused by environmental stresses [28, 29]. Nevertheless, *So* gene has an essential role in the hyphal anastomosis, presumably by regulating the secretion or perception of an undefined chemoattractant in an oscillatory manner with the *Fus3* (orthologous to MAK-2 in *N. crassa*) from the MAPK pheromone response pathway, as demonstrated for *N. crassa* [11]. Beyond *N. crassa*, the *So* was also characterized in other model organisms, plant pathogens, and endophytic fungi [15–17, 29, 30]. Though all *So* mutants fail to undergo hyphal fusions, the distinct effects on pathogenicity reported for those null mutants suggest that the biological contribution of anastomosis might depend on the infection strategies developed by different fungal pathogen species.

*Zymoseptoria tritici* is an apoplastic pathogen with a hemibiotrophic lifestyle and considered the most damaging pathogen of wheat in Europe [31]. This fungus has the ability to undergo morphological transitions in response to the environment, switching between hyphal growth and yeast-like growth [32–34]. Hyphae formed from either germinated ascospores (sexual spore), pycnidiospores (asexual spores), or blastospores (asexual yeast-like spores produced by budding) are essential for penetrating wheat leaves through the stomata and colonization of the apoplastic space. After a long asymptomatic phase (which varies depending on the wheat genotype and fungal strain combination) [35–37], the onset of the necrotrophic phase is followed by the appearance of lesions, disintegration of host tissue, and formation of asexual fruiting bodies. Though *Z. tritici* is among the top 10 most studied phytopathogens [38], there is little known about vegetative cell fusion in this organism. To date, it was shown that the deletion of the β-subunit of the heterotrimeric G protein *MgGpb1* or *ZtWor1*, a transcriptional regulator of genes located downstream of the cyclic adenosine monophosphate (cAMP) pathway, results in germ tubes that undergo extensive anastomosis [39, 40].

In this study, we aimed to determine whether vegetative cell fusions play essential biological roles in the lifestyle of *Z. tritici*. We showed that the ubiquitous ability of *Z. tritici* to undergo self-fusion was disrupted by the deletion of *ZtSof1*, affecting both hyphal and germling fusions. The characterization of mutants lacking the MAPK-encoding *ZtSlt2* or *ZtFus3* indicates a conserved role of the CWI and pheromone response pathways on fungal anastomosis*.* We found that *ZtSof1* contributes to vegetative growth and is required for melanization, but not to maintain the fungal cellular integrity. We discovered that anastomoses are dispensable for pathogenicity, but they are essential for asexual fruiting body development. In the absence of cell fusions, *Z. tritici* does not undergo asexual reproduction. These findings illustrate the impact of *ZtSof1* for fungal development and the importance of vegetative cell fusions for fungal fitness.

## Results

### Cell fusions in *Z. tritici* allow the bidirectional transfer of cytoplasmic content but do not enable multinucleated cell formation

**Fig. 1:**
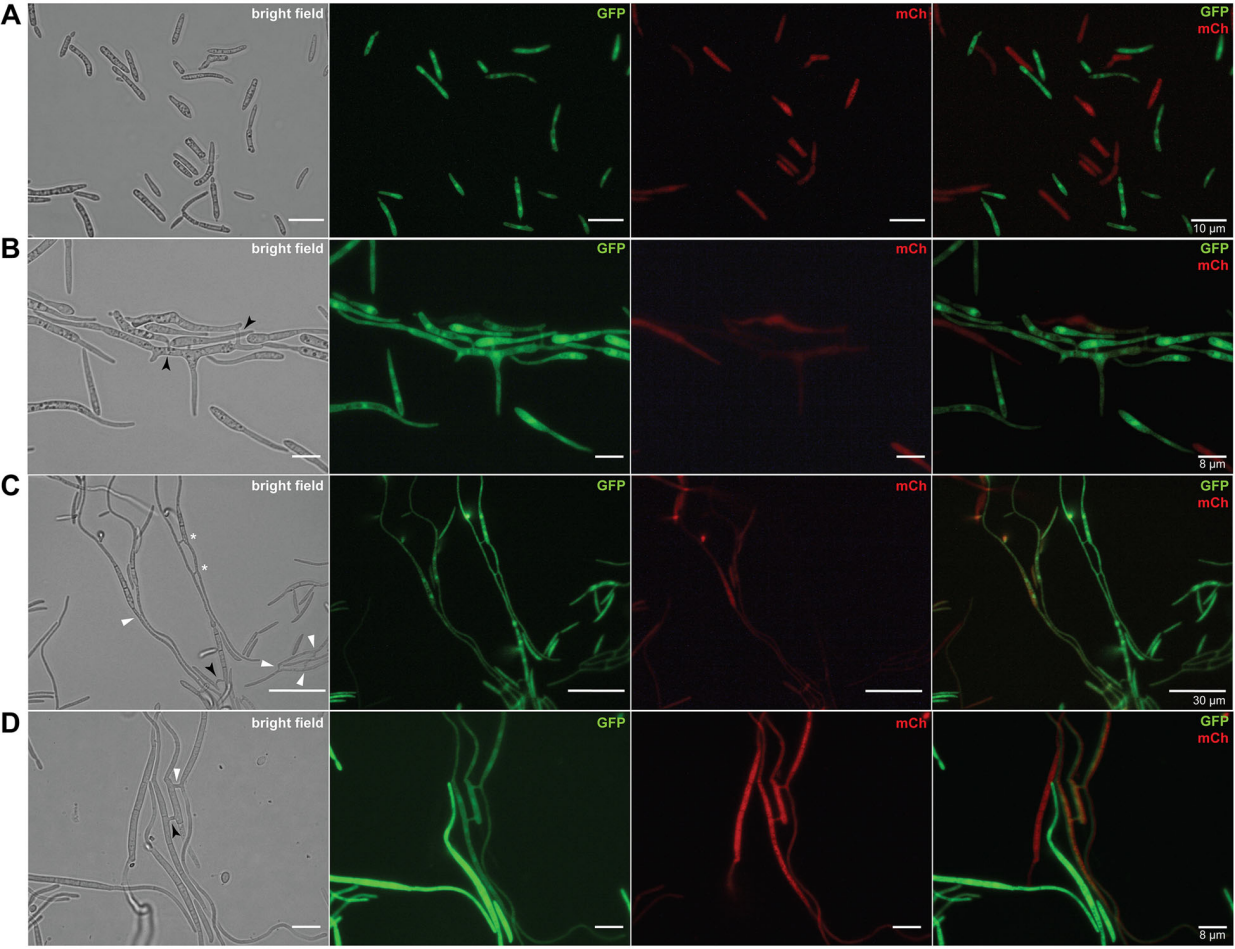
Vegetative cell fusions of *Zymoseptoria tritici*. **a** Blastospores of a GFP- or mCh-tagged 1E4 strain appeared only in a one-color channel of the fluorescence microscope. **b** High initial cell density (1 × 10^7^ blastospores/mL) induced conidial anastomosis tubes (CATs) that were frequently observed after 17 h of incubation. **c**, **d** Vegetative hyphal fusions (VHFs) from germinating blastospores or pycnidiospores of a GFP- or mCh-tagged 1E4 strain were induced at lower initial cell density (1 × 10^6^ blastospores/mL) and were noticed after 40 h of incubation. Cell fusions may result in the continuous mixing of cytoplasmic content of individuals expressing GFP or mCh fluorescent proteins. Black arrows or white triangles point to the CATs or VHFs, respectively. White asterisks point to the fusion bridges formed between individuals expressing identical fluorescent proteins

We co-inoculated either blastospores or pycnidiospores of both 1E4_GFP_ and 1E4_mCh_ fluorescent strains onto water agar (WA—1% agar in water), a hyphal-inducing medium, to investigate the ability of *Z. tritici* to undergo self-fusions. Though *Z. tritici* produces blastospores and pycnidiospores as asexual spores instead of conidium, we used the CAT terminology to define the fusion between germinating spores. CATs formed between blastospores or pycnidiospores germlings happened at high cell density (10^7^ blastospores/mL), starting after 4 h of incubation, but they were frequently observed after 17 h of incubation, resulting in cells co-expressing both fluorescent proteins (Fig. 1b). On the other hand, vegetative hyphal fusions (VHFs) from germinated blastospores or pycnidiospores were noticed at 40 h after incubation (hai) only at low cell density (10^6^ blastospores/mL) (Fig. 1c, d). Multiple interconnections via fusion bridges were observed in all tested morphotypes (Fig. 1b–d). The co-infection of wheat plants using either blastospores or pycnidiospores of 1E4_GFP_ and 1E4_mCh_ strains also resulted in VHFs on the wheat leaf surface (Additional file 1: Fig. S1). Self-fusions and cytoplasmic mixing occurred in the first 48 hai (Additional file 1a-b: Fig. S1a-b). To monitor for nucleus movement enabling multinucleated cell formation, we used the IPO323 ZtHis1-ZtGFP strain [41]. As previously reported, only one nucleus per cell was observed in this strain. No multinucleated septal compartments were observed between the two interconnected hyphae at the fusion bridges or outlying of the fusion points (Additional file 2: Fig. S2). These findings suggest that cell fusions culminate in cytoplasmic mixing in *Z. tritici*, but it does not lead to the generation of multinucleated cells in this uninucleated fungus.

### The mutual perception or response to genetically identical fusion partners requires the *ZtSof1* gene and different MAPK pathways

**Fig. 2:**
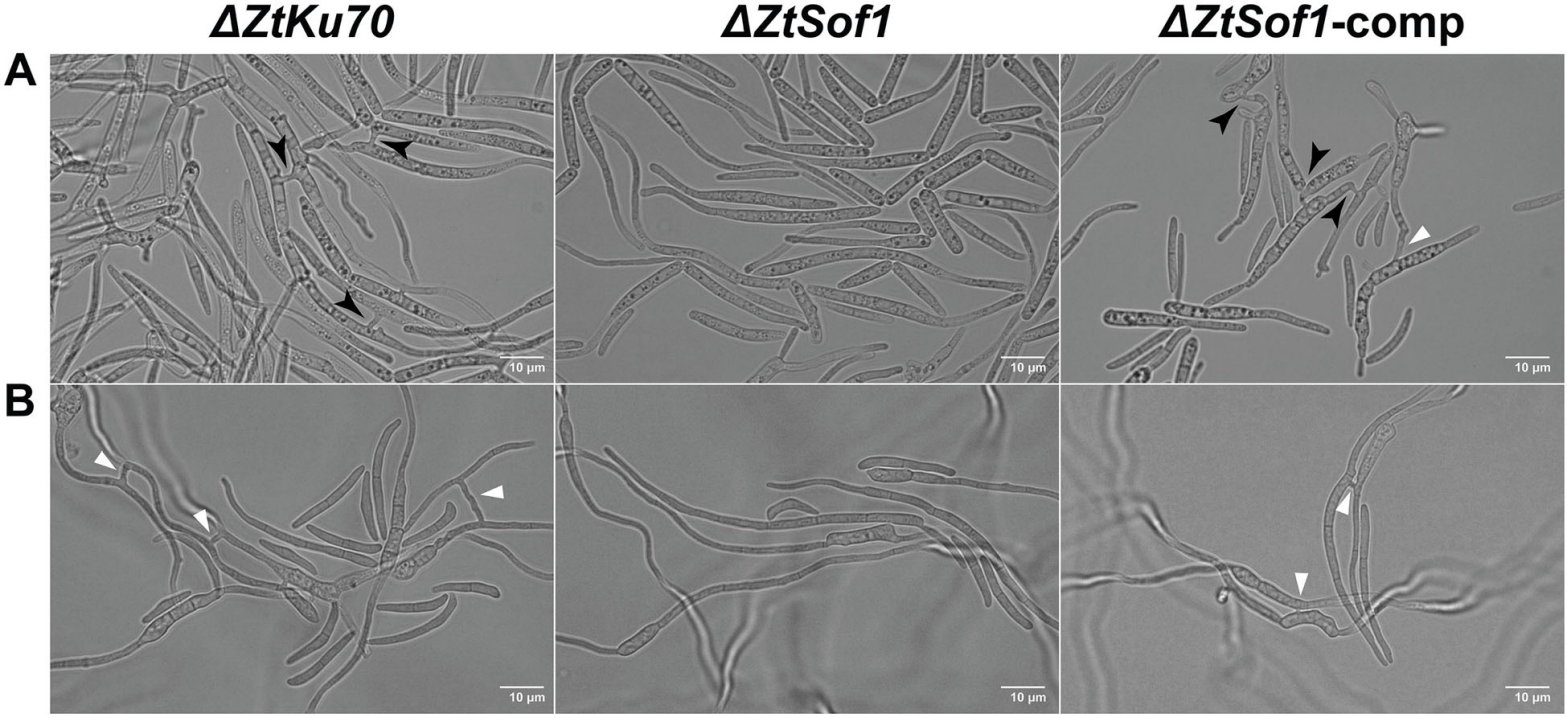
*ZtSof1* is required for vegetative cell fusion in *Zymoseptoria tritici*. All mutant lines derived from the 1E4 strain. **a** Fusion bridges between blastospore germlings were observed at high cell density for *ΔZtKu70* and *ΔZtSof1*-comp strains but not in the *ΔZtSof1* mutant. Black arrows indicate CATs. **b** VHFs were noticed at low cell density and only for those strain possessing the *ΔZtSof1* gene. White triangles point to the fusion bridges between two fused hyphal cells

*So* has an essential role in self-anastomosis [24]. To determine whether this gene plays the same role in *Z. tritici*, we identified the *So* orthologous (*ZtSof1*) in the *Z. tritici* genome (*Mycgr3G74194* or *Zt09_7_00503*), which consists of a 3794-bp open reading frame that encodes a polypeptide of 1227 amino acid and is widely distributed within the *Dothideomycetes* (Additional file 3: Fig. S3). We phenotyped the *ΔZtKu70*, *ΔZtSof1*, and *ΔZtSof1*-comp mutants generated on the 1E4 strain genetic background for the presence of interconnected individuals through anastomoses. Fusion bridges between blastospore germlings and filamentous hyphae were only observed for those strains possessing the *ZtSof1* gene, but none between *ΔZtSof1* mutant cells (Fig. 2a and b), demonstrating that *ZtSof1* plays an essential role in anastomosis between genetically identical *Z. tritici* strains.

To ensure the failure of cytoplasmic exchange on those individuals lacking the *ZtSof1* gene, we mixed blastospores of each tested strain with 1E4_GFP_ blastospores in a 1:1 ratio and traced the spores up to 40 h. Hyphal fusions and the continuous streaming of cytoplasmic green fluorescence coming from the fusion with the 1E4_GFP_ strain were observed for all tested combinations (Additional file 4: Fig. S4, and Additional file 5: Fig. S5), except for the combination of *ΔZtSof1* and 1E4_GFP_ spores (Additional file 6: Fig. S6). These findings confirmed that *ZtSof1* is required in both fusion partners for a mutual recognition previous to the anastomosis.

To determine whether the CWI and pheromone response pathways also contribute to anastomoses in *Z. tritici*, we incubated the knocked-out *ZtSlt2* (orthologous to *MAK-1* in *N. crassa*) and *ZtFus3* (orthologous to *MAK-2* in *N. crassa*) mutants on WA plates. No hyphal fusion was observed for both mutants. Unlikely, fusion bridges were regularly found between IPO323 wild-type or *ΔZtSlt2-*complemented fungal cells (Additional file 7: Fig. S7). These results indicate the recruitment of both CWI and pheromone response MAPK signaling cascades for the regulation of self-fusion in *Z. tritici*, as it has been observed in *N. crassa* [11].

### Deletion of *ZtSof1* affects growth and melanization

To assess whether vegetative cell fusions affect fungal development, we determined the radial growth of *ΔZtKu70*, *ΔZtSof1*, and *ΔZtSof1*-comp on different nutritional environments inducing different morphotypes. We observed a slightly reduced growth of *ΔZtSof1* colonies when grown on WA plates, a condition that induces hyphal growth, compared to those possessing the *ZtSof1* gene (Additional file 8a: Fig. S8a). On average, the colony radii were 5.31 ± 0.12 for *ΔZtKu70*, 4.87 ± 0.10 for *ΔZtSof1*, and 5.50 ± 0.09 for *ΔZtSof1*-comp [radial growth (mm) ± standard error] (Additional file 8c: Fig. S8c). Nevertheless, *ΔZtKu70* and *ΔZtSof1*-comp generated colonies with highly hyphal dense margins, while *ΔZtSof1* exhibited sparse filamentations at the colony periphery (Additional file 8b: Fig. S8b). Unlikely, the *ΔZtSof1* mutant grew significantly faster than the *ΔZtKu70* and *ΔZtSof1*-comp on the nutrient-rich PDA medium, a condition that induces blastosporulation (yeast-like growth) (Additional file 8e: Fig. S8e). Over time, the relative growth rate of *ΔZtKu70* and *ΔZtSof1*-comp were respectively 32% and 16% lower than the *ΔZtSof1* mutant. No morphological differences were detected between blastospores produced by the tested strains (Additional file 8d: Fig. S8d). The data suggest that *ZtSof1* mutation may affect fungal growth in a morphotype-dependent manner. Consistent with these findings, we found that the deletion of *ΔZtSof1* increased blastosporulation in a nutrient-rich liquid medium. At 48 and 72 hai, the *ΔZtSof1* mutant produced a significantly higher amount of blastospores than *ΔZtKu70* and *ΔZtSof1*-comp strains (Additional file 9: Fig. S9).

**Fig. 3:**
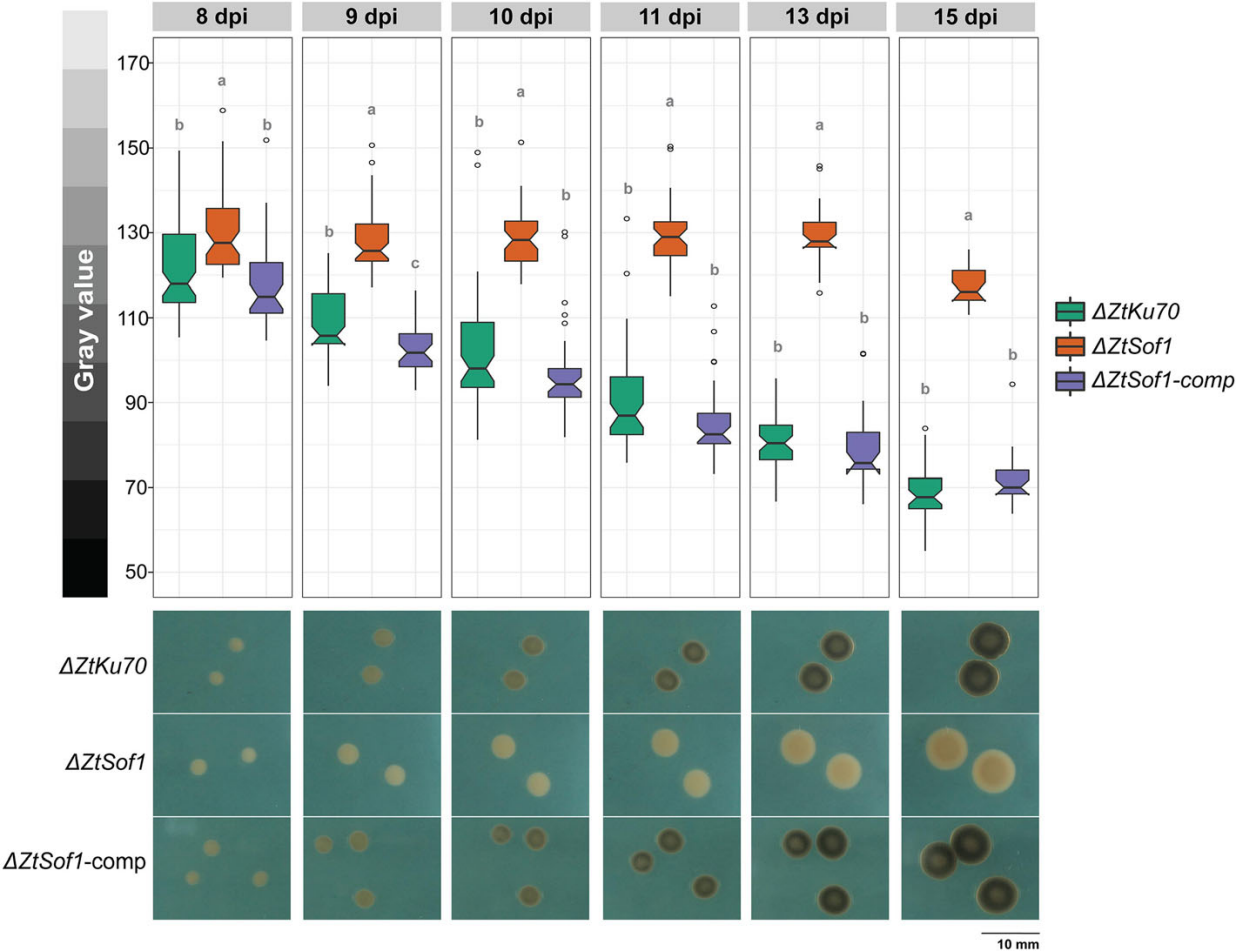
Disruption of *ZtSof1* impacts the melanization of *Zymoseptoria tritici*. The defective fusion mutant was significantly less melanized than the *ΔZtKu70* and *ΔZtSof1*-comp strains, which exhibited higher melanin accumulation over time. Bars represent standard errors of the mean gray values on at least 40 colonies. Different letters on the top of the bars indicate a significant difference among the tested strains according to the analysis of variance (ANOVA). The notch displays a 95% confidence interval of the median. Open circles represent the outlier values of each strain. Pictures shown below the bar plot represent the melanization level of *ΔZtKu70*, *ΔZtSof1*, and *ΔZtSof1*-comp strains. Gray value scale (0 = black and 255 = white) is shown on the left

**Fig. 4:**
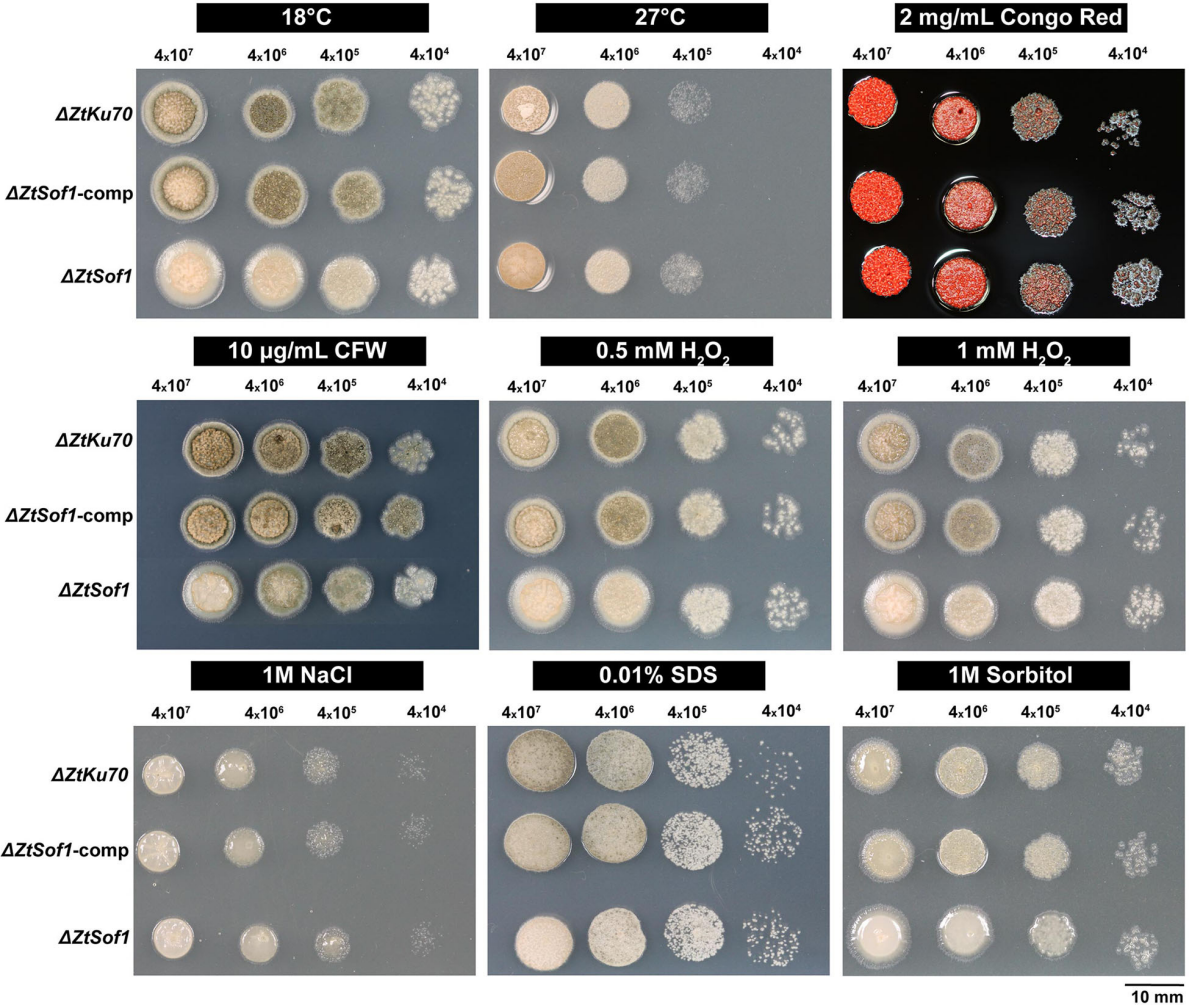
*ZtSof1* is dispensable for cellular integrity. A serial dilution of blastospore suspensions of *ΔZtKu70*, *ΔZtSof1*, and *ΔZtSof1*-comp strains were exposed for 5 days to nine different stress conditions, including different temperatures (18 °C and 27 °C), oxidative stress (0.5 and 1 mM of hydrogen peroxide (H_2_O_2_)), osmotic stresses (1 M sodium chloride (NaCl) and 1 M sorbitol), cell wall stresses (2 mg/mL Congo red and 10 μg/mL Calcofluor white (CFW)), and plasma membrane stress (0.01% sodium dodecyl sulfate (SDS)). The tested strains do not vary on their tolerance to different cellular stressors

Interestingly, no melanin accumulation was observed in *ΔZtSof1* mutant colonies (Fig. 3), demonstrating the impact of *ZtSof1* deletion on *Z. tritici* pigmentation. We postulated that the *ΔZtSof1* mutant could either display cellular integrity defects and/or being susceptible to environmental stresses. We tested nine different abiotic stressors, such as temperature, oxidative, osmotic, cell wall, and cell membrane stresses. Overall, no variability in stress responses was noticed among the strains (Fig. 4). However, *ΔZtSof1* formed slightly bigger colonies than those from *ΔZtKu70* and *ΔZtSof1*-comp, corroborating the increased growth rate observed for this mutant on nutrient-rich medium (Additional file 8e: Fig. S8e). Therefore, we found no evidence that the *ZtSof1* gene is involved in the maintenance of the cell wall integrity of *Z. tritici*.

### Hyphal fusions are essential for the development of asexual fruiting bodies

**Fig. 5:**
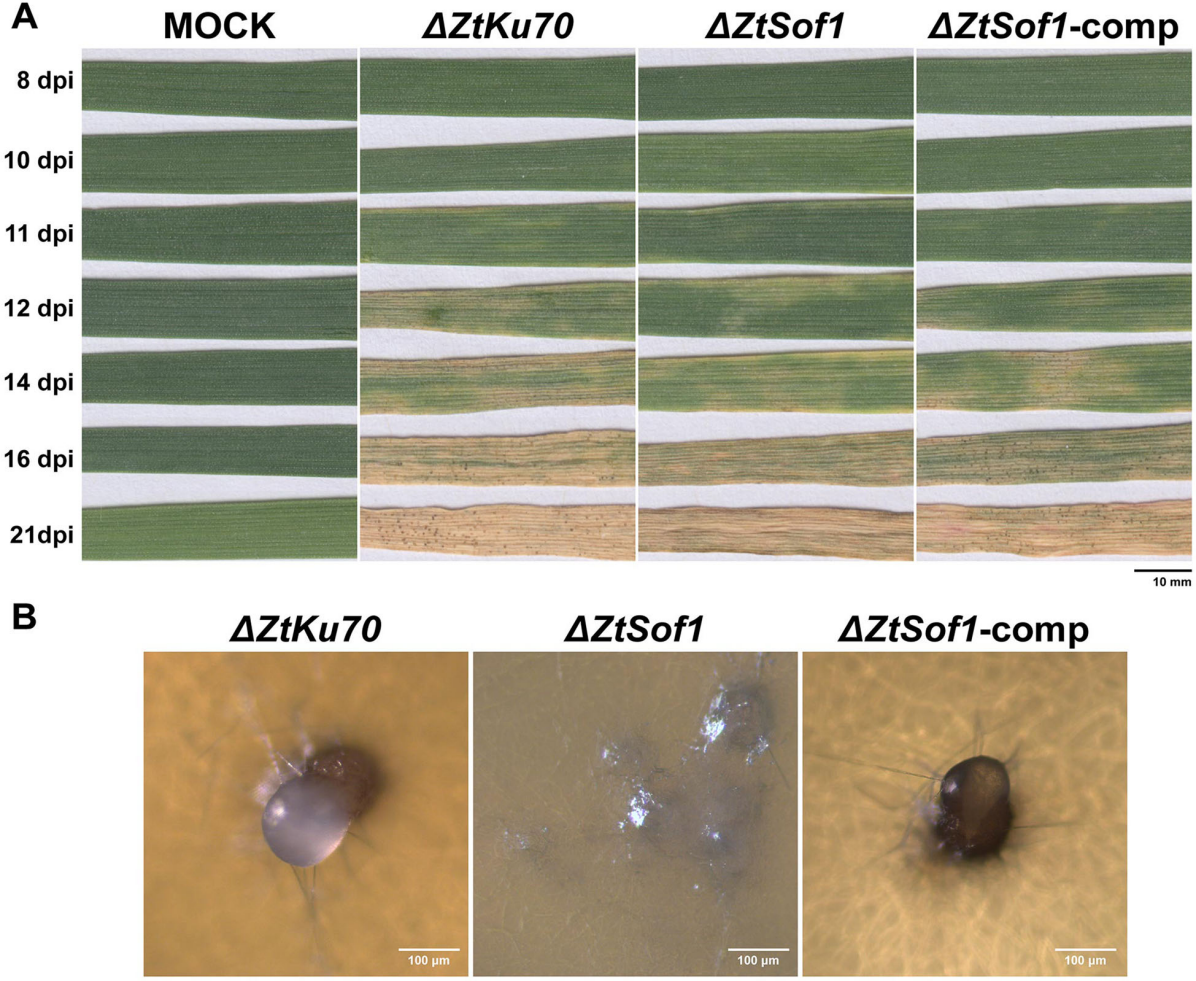
The role of vegetative hyphal fusions for disease progression and pycnidial development. **a** Susceptible wheat cultivar Drifter inoculated with *ΔZtKu70*, *ΔZtSof1*, or *ΔZtSof1*-comp strains were evaluated up to 21 days post-infection (dpi). All tested strains exhibited similar disease progression, including the onset of the necrotrophic phase at 11 dpi. **b** Pycnidia production on wheat extract agar. After 20 days of incubation, *ΔZtKu70* and *ΔZtSof1*-comp strains produced brown pycnidium-like structures exuding a whitish liquid similar to the oozed cirrhus-containing pycnidiospores spores observed for *Z. tritici*-infected wheat plants*.* In contrast, *ΔZtSof1* mutant formed mycelial knots, but those structures never developed in mature asexual fruiting bodies

We inoculated a susceptible wheat cultivar with the tested *Z. tritici* strains to assess the biological role of vegetative cell fusion during the pathogen lifecycle *in planta*. Typical symptoms caused by *Z. tritici* infections were visible after 11 days post-infection (dpi), and the disease progression was similar among the plants inoculated with *ΔZtKu70*, *ΔZtSof1*, or *ΔZtSof1*-comp strains (Fig. 5a and Additional file 10: Fig. S10). This result indicates that *ZtSof1* is neither required for host penetration nor for the asymptomatic or necrotrophic phases of the fungus. The asexual fruiting bodies (pycnidia) were visible on plants inoculated with those strains possessing the *ZtSof1* gene at 14 days post-inoculation (dpi). In contrast, plants infected with *ΔZtSof1* never developed pycnidia. The failure to undergo asexual reproduction was observed in a broad range of wheat genotypes (Additional file 11: Fig. S11).

**Fig. 6:**
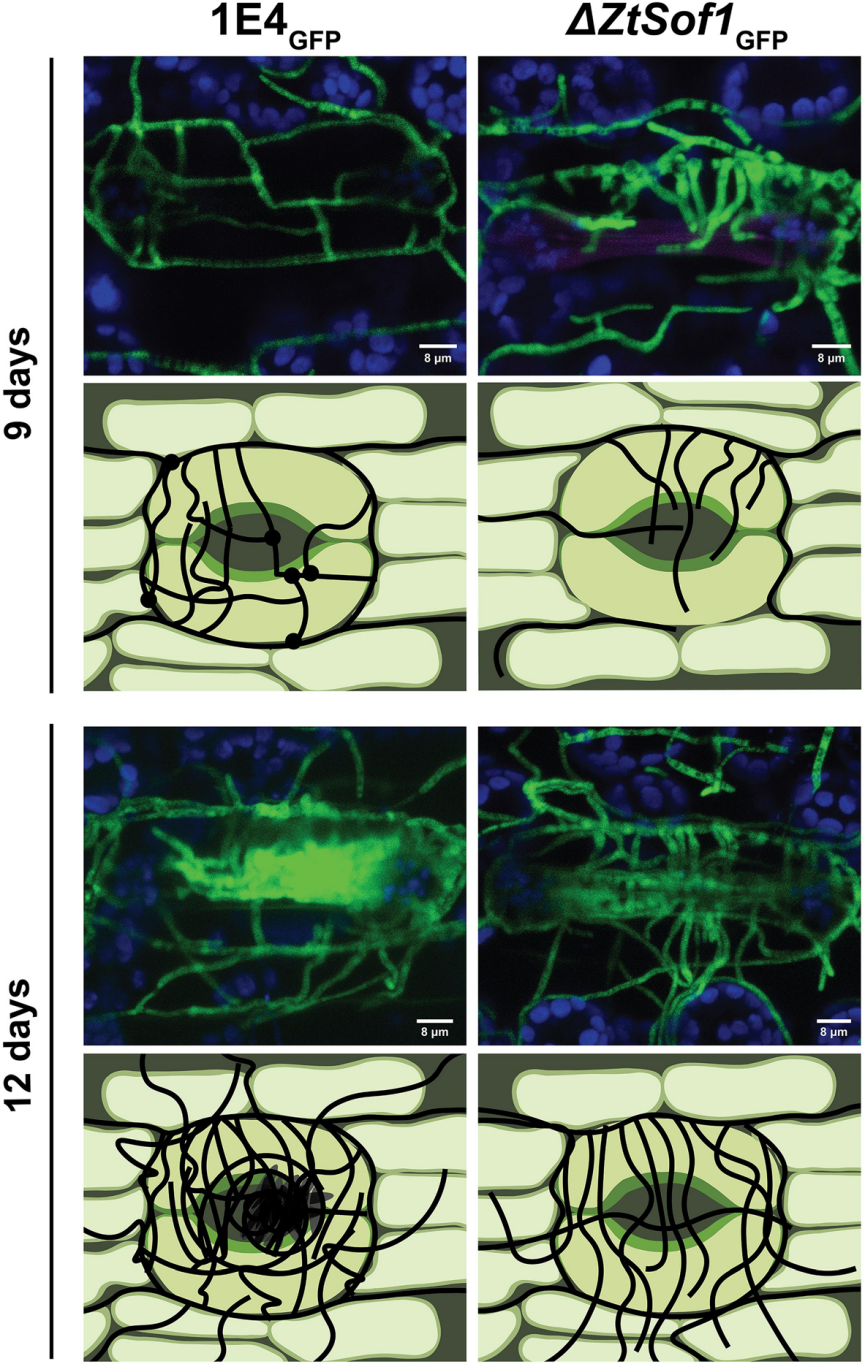
Confocal microscopy images and schematic demonstration of pycnidial development during plant infection. Susceptible wheat cultivar Drifter inoculated with the fluorescent 1E4_GFP_ (wild-type, left) or *ΔZtSof1*_GFP_ (right) strains were monitored by confocal microscopy at different days post-infection (dpi). For 1E4_GFP_, the primary intracellular hyphae surrounding the stomatal guard cells produced specialized knots from where secondary hyphae emerge and elongate. After 9 days of infection, these secondary hyphae fuse with another nearby hypha (represented by black circles), creating an interconnected network in the sub-stomatal cavity. The combination of sub-stomatal hyphal accumulation and anastomoses generates the pre-pycnidium at 12 days, which later supports the asexual reproduction of *Z. tritici*. For the ΔZtSof1_GFP_ mutant, the filamentous hyphae kept extending as individual hyphae, and no fusion points were observed. The lack of anastomosis stops the developmental process of the pycnidium formation. For earlier time points (6, 7, and 8 dpi), please see Additional file 12: Fig. S12

To distinguish whether pycnidium formation would be a consequence of the lack of hyphal fusions or susceptibility of *ΔZtSof1* cells to defense compounds produced by the plant, we used a wheat extract agar medium to induce pycnidium formation in vitro*. ΔZtSof1* aggregated into mycelial knots, which is the initial developmental stage of the pycnidium, but no asexual reproductive structures were further developed. The pycnidium-like structures formed by *ΔZtKu70* or *ΔZtSof1*-comp strains were exuding a whitish liquid containing pycnidiospores (Fig. 5b). Next, we evaluated the pycnidium formation *in planta*. The wheat plants infected with 1E4_GFP_ or 1E4_GFP_*ΔZtSof1* strains were monitored using confocal microscopy up to 12 dpi. We observed mainly host penetration, initial intercellular hyphal extension, and sub-stomatal colonization at the earlier stages of the plant infection (Additional file 12: Fig. S12 – 6 and 7 dpi). No difference was noticed in fungal development; however, hyphal fusions established during epiphytic host colonization were only detected for 1E4_GFP_ strain. At 8 and 9 dpi, we observed that the primary intercellular hyphae surrounding the stomatal guard cells produced specialized knots from where secondary hyphae emerged and elongated (Fig. 6 and Additional file 12: Fig. S12). These secondary hyphae fused with other nearby hyphae, creating an interconnected network in the sub-stomatal cavity (e.g., for the 1E4_GFP_ strain) or they kept extending as individual hyphae (e.g., for the 1E4_GFP_*ΔZtSof1* mutant) (Fig. 6 and Additional file 12: Fig. S12). The combination of sub-stomatal hyphal accumulation and anastomoses generates the mature pycnidium, which supports the asexual reproduction of *Z. tritici*. On the other hand, the lack of anastomosis stopped the symphogenous development by affecting the interweaving of hyphal branches through hyphal fusion, a crucial mechanism for the formation of pycnidia, and hence, impairing the asexual cycle of the fungus. Therefore, we concluded that hyphal fusions are decisive for the pycnidial development, and the disturbance of this mechanism ceases the asexual reproduction of *Z. tritici*.

## Discussion

The evolution of the fungal language allowed a fine-tune coordination of signal senders and receivers driving their ecological diversifications and, consequently, the regulation of complex signaling networks. Here, we explored the functional relationship of a gene involved in cell-to-cell communication and its biological contribution to the development and fitness of a fungal plant pathogen.

Vegetative cell fusion is one of the most important developmental processes of a mycelial fungal colony [10, 42]. The cytoplasmic continuity generated by cell fusion provides adaptative advantages to the interconnected mycelial network by allowing resource sharing and introgression of genetic material [19, 42, 43]. CATs forming at earlier stages of the vegetative growth have been reported in several filamentous fungi [18]. We observed that these specialized fusion bridges require a specific cell density, indicating that *Z. tritici* may induce CATs at a critical concentration of a self-produced molecule, presumably a quorum-sensing molecule. For instance, *Fusarium oxysporum* secretes and senses both a- and α-pheromone, and their perception regulates spore germination in a cell density-dependent manner [44]. On the other hand, the induction of VHFs was often observed at lower cell density, indicating that VHFs may be induced at low self-produced molecule concentration or some other environmental signal. *Z. tritici* is a pleomorphic fungus changing growth morphology according to the environment. Nutrient-limited conditions induce both hyphal growth and fusions. Plant pathogens typically experience nutrient limitations while growing on leaves, and the perception of a nutrient-limited environment may act as a stimulus to induce interconnected mycelial formation in foliar plant pathogens, as observed for the causal agent of anthracnose disease, *Colletotrichum lindemuthianum* [45]. Both germinating blastospores or pycnidiospores underwent CATs and VHFs in vitro and *in planta*, supporting our previous observation [34]. Therefore, we demonstrated that vegetative cell fusions are a ubiquitous cellular process, which may have an essential contribution to the lifestyle of *Z. tritici*.

Nucleus transfer between two encountering hyphae occurs in some fungal species [46, 47]. The consequences of genetic exchange include the formation of viable heterokaryons and the risk of introgression of pathogenic elements or virulence genes [20, 48, 49]. Unlike the majority of fungal species that form a multinucleated hyphal network [47], *Z. tritici* has only one nucleus per septal compartment [41], which raises the question of whether anastomosis would enable multinucleated cell formation in *Z. tritici*. We showed that the new cell compartment is occupied by a migrating nucleus coming from a neighboring nucleus divided by mitosis after the fusion bridge between two identical *Z. tritici* cells. However, cells containing multiple nuclei were not observed neither near nor far from the anastomosis point, indicating that *Z. tritici* may have evolved to limit the spread of genetic elements and restrict the formation of multinucleated cells. Hence, we cannot discard that multinucleated, homokaryotic, or heterokaryotic cells can be formed at low frequency in nature. These properties could impact the evolution of the pathogen, and therefore, additional studies will be needed to underlie the roles played by the genetic exchange during vegetative cell fusions in *Z. tritici*.

In the last decades, the identification of fusion-defective mutants has contributed to the understanding of the molecular mechanisms underlying cell communication and fusion, especially by unveiling the interplay of the mitogen-activated protein kinase (MAPK) pathways during chemotropic interactions [11]. MAPK pathways are involved in extracellular signal perception and regulation of diverse genes essential for mating, filamentation, pathogenicity, cell integrity, and stress responses [50–55]. Therefore, the deletion of MAPK-encoding genes results in pleiotropic phenotypes due to their involvement in multiple biological processes. Though fungal communication and fusion require the regulation of several genes [6], the cross-talk between cell wall integrity (CWI) and pheromone response, two conserved MAPK signaling pathways, is essential to produce, secrete, and sense the chemoattractant molecule produced during cell fusion [11]. The deletion of *Slt2* or *Fus3* orthologous genes disrupts the signaling cascade affecting self-anastomosis in filamentous fungi. We used the *ZtSlt2* and *ZtFus3* to demonstrate the functional conservation of these MAPK pathways in *Z. tritici*. *ZtSlt2* and *ZtFus3* are known as essential genes for the pathogenicity and developmental processes of *Z. tritici*, including vegetative growth, melanization, and pycnidium formation [56, 57]. Here, we showed that both *ΔZtSlt2* and *ΔZtFus3* mutants were unable to undergo anastomosis. Our results indicate that cell fusions in *Z. tritici* follow the same signaling mechanism described for *N. crassa* [11, 58], where the signal sending and receiving are coordinated by genes associated with the CWI and pheromone response pathways. Besides, this is the first report of those pathways regulating cell communication in *Z. tritici*.

To evaluate the impact of vegetative cell fusion in the biology of a hemibiotrophic fungus, we used the *ZtSof1* orthologous of the *N. crassa So* gene. Contrary to the MAPK-related genes, the characterization of *So* orthologs results in low pleiotropy [15, 16, 24]. Filamentous fungi lacking *So* gene were impaired in self-anastomosis [15–17, 24], including *Z. tritici*, in which the deletion of *ZtSof1* abolished vegetative cell fusion. We characterized the impact of the fusion deficiency at different developmental stages of *Z. triti*ci. For instance, the *ΔZtSof1* mutant exhibited a more asymmetrical hyphal extension in a nutrient-poor medium inducing hyphal growth than the fusion-competent individuals. It was demonstrated that the direction of the nutrient distribution occurs from the central part of the mycelium to outwards, and its streaming speed is driven by anastomosis [42]. Thereby, the reduced colony extension observed for the *ΔZtSof1* may be a consequence of the irregular distribution of cytoplasmic content throughout the mycelial colony. In contrast, the induced blastosporulation and larger yeast-like colonies exhibited by the fusion-defective mutant in nutrient-rich environments may be a consequence of the disruption of the CWI pathway or its cross-talk with other MAPK pathways involved in fungal growth. The genetic relationship between vegetative growth and the *ZtSof1* gene remains to be elucidated.

We showed that the *ΔZtSof1* colonies do not accumulate melanin, resulting in whiter and larger colonies than those formed by *ΔZtKu70* and *ΔZtSof1*-comp strains. These findings suggest a possible trade-off between energy cost for pigment production and growth. The reduction of fungal growth caused by the higher accumulation of melanin was reported before for *Z. tritici* [59]. Melanins are dark-pigmented secondary metabolites often associated with the fungal cell walls. Though fungi can produce different kinds of melanins, it is suggested that melanization of *Z. tritici* is only controlled by the polyketide synthase (PKS) gene cluster containing catalytic enzymes and transcription regulators of the 1,8-dihydroxynaphthalene (DHN) melanin [59, 60]. It was demonstrated for plant pathogenic fungi that the deletion of CWI-associated genes inhibited pigmentation by reducing the expression of DHN melanin biosynthetic genes [61–64]. Therefore, the deletion of *ZtSof1* may impair the CWI-regulatory cascade and, consequently, the regulation of DHN-melanin production, resulting in the lack of pigmentation observed for both *ΔZtSof1* and *ΔZtSlt2* [56] mutants. On the other hand, the CWI, high-osmolarity glycerol (HOG), cyclic adenosine monophosphate (cAMP), and the pheromone response pathways can interact regulating melanization in a cooperative manner [65–67]. Consistent with the findings in other fungi, mutants in these signaling pathways, including *ΔZtFus3*, display altered pigmentation in *Z. tritici* [33, 39, 56, 57, 68, 69]. Additionally, a QTL mapping study identified *ZtSlt2*, *ZtHog1*, *ZtGpa1*, and *ZtFus3*, contributing to the melanization of this fungus [60]. Thereby, we cannot discard that the deletion of *ZtSof1* may affect the cross-talk between CWI with other signaling cascades impairing fungal melanization. Further investigations are needed to elucidate both hypotheses.

Melanin is postulated to contribute to fungal protection against fungicide and environmental stresses in *Z. tritici* [59, 60], and its regulation depends on environmental cues and colony development [60], though it is not yet a fully understood mechanism. We used nine different cellular stressors to evaluate whether (i) the defect in melanin accumulation or (ii) the deletion of *ZtSof1*, the scaffold protein for the MAPK genes from the CWI pathway, affects pathogen stress tolerance. Since the non-melanized *ΔZtSof1* mutant displayed the same degree of stress sensitivity than *ΔZtKu70* and *ΔZtSof1*-comp strains, we concluded that *ZtSof1* does not act as a scaffold protein for all CWI pathway functions. Consistent with our findings, the model fungus *Sordaria macrospora*, the PRO40 (orthologous to *ZtSof1*), operates as a scaffold for the CWI-encoding genes during fungal development, hyphal fusion, and stress response, but not for growth under cell wall stress agents [25]. Our results showed that melanin accumulation of *Z. tritici* does not benefit fungal survival in harsh environments, at least for the stressful conditions tested in this study.

We demonstrated that VHFs are dispensable for the pathogenicity of *Z. tritici*. The fusion-defective *ΔZtSof1* mutant displayed a similar host damage progression than those individuals possessing the gene. This finding exemplifies the distinct effects of cell fusions on fungal pathogenicity. For instance, for the soil-borne *Fusarium oxysporum*, VHF-impaired mutants exhibited only a slightly reduced virulence, whereas for the necrotrophic plant pathogen *Alternaria alternata*, VHFs are necessary for the full virulence of the fungus [15, 16]. Though the deletion of *ZtSof1* is not essential for host penetration, colonization, or for the onset of the necrotrophic phase *per se*, it is during hyphal accumulation in the sub-stomatal cavity that the fusion defect impacts *Z. tritici* fitness. The infection process during wheat colonization was microscopically detailed before for *Z. tritici* [70]; however, the study did not explore the contribution of VHFs. Here, we showed that the primary intercellular hyphae surrounding the stomatal guard cells produced specialized knots from where secondary hyphae emerge and elongate to fuse with other adjacent hyphae. Hence, the preliminary hyphal network creates the basis for a symphogenous development that builds the concave-shaped pycnidial wall of the mature pycnidium. On the other hand, the inability to undergo anastomosis ceases the development of the asexual fruiting bodies at this stage, and therefore abolishes fungal reproduction. The accumulation of hyphae observed in the sub-stomata chamber by the *ΔZtSof1* mutant confirms that a specific signal triggers sub-stomatal hyphal aggregation independent of the putative chemoattractant molecule secreted by the fungus to induce hyphal fusion. In line with this hypothesis, a recent study showed that the disruption of the transcription factor *ZtStuA* impairs hyphal aggregation, resulting in the lack of pycnidium formation in vitro and *in planta* [68]. Therefore, we suggest that *ZtStuA* acts regulating genes necessary for the pre-pycnidium stages (e.g., hyphal aggregation) [68], while *ZtSof1* controls the hyphal fusions required for the formation of the mature pycnidium. The initiation of the asexual sporulation may depend on the regulatory genes, such as *ZtBrlA2* and *ZtFlbC*, as described by Tiley et al. Moreover, the inability of *ΔZtFus3* to undergo VHFs and produce pycnidia [57] confirms the interplay between the CWI and pheromone response pathways during hyphal fusion, a developmental process essential for the asexual reproduction of *Z. tritici*. Fleissner et al. [24] demonstrated that the deletion of *So* in *N. crassa* also affects female fertilization, blocking the sexual reproduction of the mutant. Further experiments need to be performed to address this question for *Z. tritici*, but, likely, the *ZtSof1* gene may also play a crucial role in the sexual reproduction of this pathogen.

## Conclusion

The characterization of the *ZtSof1* gene demonstrated its fundamental role in fungal biology. Beyond the impact of *ZtSof1* for self-fusion, we show the contribution of this gene for fungal development, including vegetative growth and melanization. Besides, we demonstrated that VHFs are dispensable for pathogenicity, but essential for pycnidial development, and these mechanisms might be controlled by the interplay between the CWI and the pheromone response pathways. Our data show how cell fusion affects *Z. tritici* fitness and provides a new gene target to control septoria tritici blotch (STB) disease.

## Methods

### Strains and growth conditions

The Swiss *Z. tritici* strain ST99CH_1E4 (abbreviated as 1E4), described by Zhan et al. [71], and mutant lines derived from this strain were used in this study. 1E4 strains expressing cytoplasmic GFP (1E4_GFP_) or mCherry (1E4_mCh_) were provided by Andrea Sanchez-Vallet after being generated by Sreedhar Kilaru and Gero Steinberg. The knocked-out *ΔZtSlt2* [56] and *ΔZtFus3* [57] mutants were provided by Marc-Henri Lebrun (National Institute of Agricultural Research – INRA, France). Because the MAPK mutants were generated in the genetic background of IPO323 [72], this strain was also used as a control. The strain IPO323 ZtHis1-ZtGFP [41] was provided by Gero Steinberg (Exeter University). Each strain was stored in glycerol at − 80 °C until required and then recovered in yeast-sucrose broth (YSB) medium (10 g/L yeast extract, 10 g/L sucrose, 50 μg/mL kanamycin sulfate; pH 6.8) incubated at 18 °C for 4 days.

### Plant infection to obtain fluorescent pycnidiospores

Wheat seedlings from the susceptible wheat cultivar Drifter were grown for 16 days in the greenhouse at 18 °C (day) and 15 °C (night) with a 16-h photoperiod and 70% humidity. Blastospore suspensions of 1E4_GFP_ or 1E4_mCh_ were obtained after 4 days of growth in the YSB medium. Spore suspensions were adjusted to a final concentration of 10^6^ blastospores/mL in 30 mL of sterile water supplemented with 0.1% (v/v) Tween and applied to run-off using a sprayer, and the plants were kept for 3 days in sealed plastic bags, followed by 21 days in a greenhouse. Leaves with pycnidia were harvested and transferred to a 50-mL Falcon tube containing sterile water and gently shaken to harvest the pycnidiospores. Fluorescent pycnidiospores were used to assess the cell fusion events during in vitro and in vivo growth. Pycnidiospore suspension-tagged GFP or mCherry were also adjusted to a final concentration of 10^6^ pycnidiospores/mL, and a new batch of plants was inoculated as described above. Plants co-infected by both 1E4_GFP_ and 1E4_mCh_ strains were used to observe VHFs on the wheat leaf surface.

### Characterization of cell fusion events in vitro and in vivo

The ability of *Z. tritici* to undergo cell fusions was evaluated using blastospores and pycnidiospores of 1E4_GFP_ and 1E4_mCh_. Cell concentrations were adjusted to 3.3 × 10^7^ blastospores/mL or 3.3 × 10^6^ blastospores/mL to induce CATs or VHFs, respectively. Three hundred microliters of each morphotype and fluorescence was plated on WA to create a ratio of 1:1 and to provide a final concentration of 10^7^ blastospores/mL or 10^6^ blastospores/mL. A section of about 1 cm^2^ of agar was aseptically cut and placed on a microscope slide. The mixing of both cytoplasm contents through CATs or VHFs was checked up to 40 hai using a Leica DM2500 fluorescent microscope with LAS v.4.6.0 software. GFP excitation and emission was at 480/40 nm and 527/30 nm, respectively, whereas mCherry was excited at 580/20 nm and detected at 632/60 nm.

VHFs during spore germination on wheat leaf surfaces were obtained via confocal images using a Zeiss LSM 780 inverted laser scanning microscope with ZEN Black 2012 software. An argon laser at 500 nm was used to excite GFP fluorescence and chloroplast autofluorescence, while mCherry excitation was at 588 nm. The emission wavelength was 490–535, 624–682, and 590–610 nm for GFP, chloroplast autofluorescence, and mCherry, respectively. Plants co-inoculated with blastospores or pycnidiospores of 1E4_GFP_ and 1E4_mCh_ strains were checked up to 48 hai.

### Plasmid constructions and transformations

Primers used for cloning, sequencing, and knock-out confirmations are listed in Table S1. DNA assemblies were conducted with the In-Fusion HD Cloning Kit (Takara BIO) following the manufacturer’s instructions. The plasmid constructions to generate the three mutants in the 1E4 genome background and used in this study (*ΔZtKu70*, *ΔZtSof1*, and *ΔZtSof1*-comp) are described in Additional file 13: Fig. S13. The pES1-*ΔZtSof1* construction was also used to knock out the *ZtSof1* gene in the 1E4_GFP_ genome background, enabling the visualization of the GFP-tagged mutant during host infection.

*Z. tritici* 1E4 strain was transformed by *Agrobacterium tumefaciens*-mediated transformation (ATMT) according to Meile et al. [73]. The knock-out of the target genes was verified by a PCR-based approach using a forward primer specific to the upstream sequence of the disrupted gene and a reverse primer specific to bind in the resistance cassette (Additional file 14: Table S1). We determined the copy number of the transgene by quantitative PCR (qPCR) on genomic DNA extracted with the DNeasy Plant Mini Kit (Qiagen). We used qPCR target gene as the selection marker and the 18S rDNA as the reference gene (Additional file 14: Table S1). Lines with a single insertion were selected for further experiments.

### Phenotypic characterizations

For all phenotypic analyses, *ΔZtKu70* was considered the wild-type (WT) strain. To pinpoint the role of the *ZtSof1* gene on the vegetative cell fusion, we added blastospore suspension cells of *ΔZtKu70*, *ΔZtSof1*, and *ΔZtSof1*-comp to a final concentration of 10^6^ blastospores/mL or 10^7^ blastospores/mL into WA and incubated at 18 °C to induce CATs or VHFs, respectively. For the MAPK *ΔSlt2* and *ΔFus3*, IPO323 and *ΔSlt2*-complemented strains, WA plates were inoculated only at a final concentration of 10^6^ blastospores/mL. Cell fusion events were monitored up to 40 hai by light microscopy. Because fusion bridges were not observed between individuals lacking *ΔZtSof1*, we mixed 150 μL of 10^6^ blastospores/mL of *ΔZtKu70*, *ΔZtSof1*, or *ΔZtSof1*-comp strains with the same concentration of 1E4_GFP_ blastospores in a ratio of 1:1 to confirm the failure of cytoplasmic streaming. At least 50 spores of each sample combination were monitored.

To test for altered fungal growth, we used PDA (39 g/L potato dextrose agar, 50 μg/mL kanamycin sulfate) or WA media to induce blastospore or hyphal growth, respectively. Two hundred microliters of spore suspension of *ΔZtKu70*, *ΔZtSof1*, and *ΔZtSof1*-comp was plated at a final concentration of 2 × 10^2^ blastospores/mL on each medium as mentioned earlier and incubated in the dark at 18 °C. At least 40 colonies formed in five independent PDA plates were photographed from the bottom using a standardized camera setting [60] at 8, 9, 10, 11, 13, and 15 days post-incubation (dpi). Digital images were processed using a macro developed in the ImageJ software [74], which scores the area of individual colonies in the images. Fungal growth was obtained by converting the colony area into radial growth (mm) based on the formula $$ =\sqrt{\begin{array}{c}\boldsymbol{A}\\ {}\boldsymbol{\pi} \end{array}} $$. Radial growth values were plotted in a boxplot graphic using the ggplot2 package from R [75]. Analysis of variance (ANOVA) was performed to determine the differences in fungal growth among the strains using the agricolae package in R [76]. The radial growth rate (mm/day) for each strain was measured by plotting the colony radius over time, which fitted to a linear model (Pearson’s correlation coefficient value (*r*^2^ ≥ 0.98)). The relative growth rate was calculated by dividing the slope of the regression line of *ΔZtSof1* by the slope of *ΔZtKu70* or *ΔZtSof1*-comp strains. To measure mycelial growth on WA, we calculated the mycelial diameter from digital images of at least 40 colonies formed in five independent Petri dishes at 15 dpi and using ImageJ software [74]. The mycelial diameter values were divided by two to generate the radial growth (mm) values.

The effect on the blastosporulation was assessed by adding each tested *Z. tritici* strain at an initial concentration of 10^4^ blastospores/mL into YSB and incubated at 18 °C. Aliquots of each flask were taken every 24 h for 4 days, and the blastospore concentration was calculated using a KOVA cell chamber system (KOVA International Inc., USA).

The degree of melanization was estimated from at least 40 colonies formed on PDA plates. We used a macro developed in ImageJ [74], which scores the mean gray value of each colony. Gray values range from 0 to 255, with 0 representing black and 255 representing white. The mean gray values of each strain over time were plotted in a boxplot using ggplot2 package from R [75].

The impact on the cell integrity caused by the deletion of *ΔZtSof1* was verified by exposing the blastospores of *ΔZtKu70*, *ΔZtSof1*, and *ΔZtSof1*-comp to nine different stress conditions, including different temperatures (18 °C and 27 °C), oxidative stress (0.5 and 1 mM of hydrogen peroxide (H_2_O_2_)), osmotic stress (1 M sodium chloride (NaCl) and 1 M sorbitol), cell wall stress (2 mg/mL Congo red and 10 μg/mL Calcofluor white (CFW)), and plasma membrane stress (0.01% sodium dodecyl sulfate (SDS)). Spore suspensions of each strain were serial diluted to 4 × 10^4^, 4 × 10^5^, 4 × 10^6^, and 4 × 10^7^ blastospores/mL, and drops of 3.5 μL were plated on five independent PDA plates amended with the mentioned stresses and incubated at 18 °C. Colony phenotypes were assessed by digital images taken at 5 dpi.

### Virulence assay and pycnidium formation in vitro

Seeding, greenhouse and plant growth conditions, inoculum preparation, and plant inoculation followed the procedures described by Meile et al. [73]. To estimate the percentage of leaf covered by lesions (PLACL) and pycnidium formation, we harvested the second leaves of Drifter plants inoculated with *ΔZtKu70*, *ΔZtSof1*, or *ΔZtSof1*-comp strains at 8, 10, 11, 12, 14, 16, and 21 dpi. Leaves were mounted on a paper sheet, scanned with a flatbed scanner, and analyzed using automated image analysis [77]. Data analysis and plotting were performed using the ggplot2 package [75].

The defect in pycnidium formation was confirmed by plating blastospores of *ΔZtKu70*, *ΔZtSof1*, and *ΔZtSof1*-comp strains onto wheat extract agar medium (50 g/L blended 21-day-old wheat leaves cultivar Drifter, 10 g/L agar) and incubated under UV-A light (16:8 light:dark cycle) up to 40 days at 18 °C, following the protocol optimized by Tiley et al. [68].

### Confocal laser scanning microscopy of infected wheat leaves

To assess the impact of *ZtSof1* deletion on fungal fitness during host colonization, we inoculated wheat plants with 1E4_GFP_ and other two independent GFP-tagged *ΔZtSof1* mutants. Infected leaves were harvested at 6, 7, 8, 9, 10, 11, and 12 dpi and checked for developmental stages of asexual fruiting bodies. Microscopy was conducted using Zeiss LSM 780 inverted laser scanning microscope with ZEN Black 2012 software. An argon laser at 500 nm was used to excite GFP fluorescence and chloroplast autofluorescence with an emission wavelength of 490–535 nm and 624–682 nm, respectively. Analyses, visualization, and processing of image *z*-stacks were performed using ImageJ software [74].

## Supplementary information

**Additional file 1: Figure S1.** Vegetative hyphal fusion occurs during epiphytic growth on wheat leaves. Co-infection of wheat plants with blastospores (A) or pycnidiospores (B) from the 1E4 strain expressing either the cytoplasmic green fluorescent protein (GFP) or the red-fluorescent protein (mCherry) resulted in hyphal fusions and cytoplasmic exchange after 48 hours of infection. Hyphal fusion during epiphytic colonization may assist the fungus to create an interconnected network supporting its establishment on the leaf surface before host penetration.

**Additional file 2: Figure S2.** Hyphal fusion does not lead to the generation of multinucleated cells in *Zymoseptoria tritici*. Blastospores of the IPO323 ZtHis1-ZtGFP strain, which has the GFP as a fluorescent marker labeling the nucleus, were plated on WA plates at a final concentration of 10^6^ blastospores/mL and incubated at 18°C. After 72 hours of incubation, none septum containing more than one nucleus was observed neither at hyphal bridges nor distant of the fusion point. Black triangles indicate the septal compartment containing only one nucleus at the fusion bridges.

**Additional file 3: Figure S3.** Scheme showing the phylogenetic relationship of the *So* gene within Ascomycete species. The *so* gene sequence from *Neurospora crassa* (XM_958983.3) was blasted against the *Z. tritici* genome (https://genome.jgi.doe.gov/Mycgr3/Mycgr3.home.html) to identify its orthologous in this fungus. The *Z. tritici* So orthologous protein sequence was used for a Blastp analysis against the NCBI database (National Center of Biotechnology Information). Blastp searches at expected value homology cut-off of 1e^-10^ were included as positive. A dataset containing So orthologous proteins of different Ascomycete species were used for phylogenetic analysis. Protein sequences were aligned using the AliView program [78]. The best-fit model of amino acid evolution was the LG+G, determined by Mega6 software [79]. Amino acid sequences were aligned using Muscle, followed by maximum likelihood (ML) phylogeny reconstruction using 1,000 bootstraps and performed with the software Mega6 [79]. (A) The illustration demonstrates the *ZtSof1* gene locus and its protein sequence containing the Atrophin 1, WW, and PhoD, as protein domains. Comparison of ZtSof1 protein sequence with its orthologs showed 53% identity with *Epichloe festucae;* 54% identity with *Neurospora crassa* and *Sordaria macrospora;* 55% identity with *Fusarium oxysporum*; 60% identity with *Aspergillus oryzae*; and 63% identity with *Alternaria brassicicola*. (B) The alignment of the WW protein-protein interaction domain, including the PPLP motif of 41 different fungal species. Red boxes surround the two conserved tryptophan residues spaced by 22 amino acids apart. (C) Phylogenetic analysis grouped the orthologs of the *ZtSof1* gene onto three groups based on fungal Classes (Dothideomycetes, Sordariomycetes, and Chaetothyriomycetes together with Eurotiomycetes), independently whether they were parasites, mutualists or saprotrophs. Three members of Basidiomycetes were used as an outgroup to root the tree.

**Additional file 4: Figure S4.** Cytoplasmic streaming between *ΔZtKu70* and the GFP-tagged 1E4 strain. Blastospores of *ΔZtKu70* and 1E4_GFP_ were co-inoculated on water agar (WA) plates, a hyphal fusion-inducing condition. After 40 hours of incubation, fusion bridges were observed between *ΔZtKu70* and 1E4_GFP_ strains. The detection of the green fluorescent protein in the cytoplasm of the recipient hypha *ΔZtKu70* confirms the cytoplasmic streaming between the two fused individuals (panel 1). Black asterisk points to the non-fluorescent ΔZtKu70 spore before hyphal fusion. White triangle indicates the fusion point between the *ΔZtKu70* and 1E4_GFP_ strains.

**Additional file 5: Figure S5.** Cytoplasmic streaming between *ΔZtSof1*-comp and GFP-tagged 1E4 strain. Blastospores of *ΔZtSof1*-comp and 1E4_GFP_ were co-inoculated on water agar (WA) plates, a hyphal fusion-inducing condition. After 40 hours of incubation, fusion bridges were observed between *ΔZtSof1*-comp and 1E4_GFP_ strains. The detection of the green fluorescent protein in the cytoplasm of the recipient hyphae *ΔZtSof1*-comp confirms the cytoplasmic streaming between the fused individuals (panel 1). Black asterisks point to the non-fluorescent *ΔZtSof1*-comp spore before hyphal fusion. White triangles indicate the fusion points between the *ΔZtSof1*-comp and 1E4_(GFP)_ strains.

**Additional file 6: Figure S6.** Co-inoculation of *ΔZtSof1* and GFP-tagged 1E4 strain confirms the failure of the *ΔZtSof1* mutant to undergo hyphal fusion. Blastospores of *ΔZtSof1* and 1E4_GFP_ were co-inoculated on water agar (WA) plates, a hyphal fusion-inducing condition. After 40 hours of incubation, fusion bridges were only observed between 1E4_GFP_ germinating spores (panel 1). Fluorescent green protein was never detected on the cytoplasm of *ΔZtSof1* cells (panels 1, 2, and 3). The filamentous of the *ΔZtSof1* mutant grew in parallel with those hyphae from the 1E4_GFP_, but they never undergo hyphal fusion (panels 2 and 3), demonstrating that *ZtSof1* is required in both fusion partners to establish the fungal communication required for perception or response during cell fusion. Black asterisks point to the non-fluorescent *ΔZtSof1* spores.

**Additional file 7: Figure S7.** MAPK-encoding *ZtSlt2* and *ZtFus3* genes are required for anastomosis in *Zymoseptoria tritici*. (A) Hyphal fusions were regularly found in the wild-type strain (IPO323). The deletion of *ZtSlt2* (orthologous to *MAK-1*) or *ZtFus3* (orthologous to *MAK-2*) resulted in fusion-defective mutants, probably due to the disruption of the oscillatory recruitment of both MAPK modules required for cell-to-cell communication and fusion, as described for *Neurospora crassa* [11]. The defective phenotype was restored in the complemented *ΔZtSlt2*-comp strain. White triangles point to self-fusion events. (B) Deletion of the MAPK *Slt2* or *Fus3* is dispensable for the cellular integrity of *Z. tritici*. A serial dilution of blastospore suspensions of IPO323, *ΔZtSlt2*, *ΔZtSlt2*-comp, and *ΔZtFus3* strains were exposed for five days to cell wall stresses (2 mg/mL Congo red - CR and 10 μg/mL Calcofluor white - CFW). The tested strains do not vary on their tolerance to the cellular stressors.

**Additional file 8: Figure S8.** *ZtSof1* impact the vegetative growth in a morphotype-depending manner in *Zymoseptoria tritici*. A nutrient-poor medium (WA), inducing hyphal growth, and a nutrient-rich medium (PDA), inducing blastosporulation, were used to assess the effect of *ZtSof1* deletion on fungal radial growth. (A) The *ΔZtSof1* mutant exhibited a similar colony morphology than *ΔZtKu70* and *ΔZtSof1*-comp strains on WA plates. (B) Light microscopy of colony edges showed a dense hyphal-thickened margin for *ΔZtKu70* and *ΔZtSof1*-comp colonies, whereas the *ΔZtSof1* mutant exhibited only a few filamentous at the colony periphery. Dashed squares point to the localization of microscope images. (C) Thought no morphological differences were observed for the tested *Z. tritici* strains, the fusion defective *ΔZtSof1* mutant had a slight, but significant reduction of its radial growth (mm) compared to *ΔZtKu70* and *ΔZtSof1*-comp when grown on a nutrient-limited medium. At least 40 colonies of each tested strains were evaluated. Two and three stars indicate a *p*-value <0.005 and <0.0005, respectively. (D) No morphological differences were noticed for the blastospores produced by *ΔZtKu70*, *ΔZtSof1*, or *ΔZtSof1*-comp strains. (E) *ΔZtSof1* mutant grew faster and had higher radial growth over time than the *ΔZtKu70* and *ΔZtSof1*-comp strains when incubated on PDA. Bars represent standard errors of the radial growth (mm) of at least 40 colonies. Different letters on the top of the bars indicate a significant difference among the tested strains according to the Analysis of Variance (ANOVA). The notch displays a 95% confidence interval of the median. Open circles represent the outlier values of each strain. Pictures shown below the bar plot illustrate the colony sizes of *ΔZtKu70*, *ΔZtSof1*, and *ΔZtSof1*-comp strains.

**Additional file 9: Figure S9.** *ZtSof1* deletion induced blastosporulation in *Zymoseptoria tritici*. The nutrient-rich YSB medium was used to access the contribution of *ZtSof1* to the blastospore formation. The experiment was performed twice with similar results.

**Additional file 10: Figure S10.** Percentage of leaves covered by lesions (PLACL). The second leaves of the wheat cultivar Drifter infected with *ZtKu70*, *ΔZtSof1*, and *ΔZtSof1*-comp strains and harvested at different days post-inoculation (dpi). Bars represent standard errors of PLACL values of at least six infected leaves. Different letters on the top of the bars indicate a significant difference among the tested strains according to the Analysis of Variance (ANOVA). Black circles represent the outlier data points.

**Additional file 11: Figure S11.** *ΔZtSof1* and *ΔZtKu70* strains do not vary in pathogenicity, except for the failure of the *ΔZtSof1* mutant to undergo asexual reproduction. Five different winter cultivars infected with *ΔZtKu70* or *ΔZtSof1* strains were, respectively, evaluated at 14- and 21 days post-inoculation (dpi) for host damage and pathogen reproduction. We used five different winter cultivars of wheat (*Triticum aestivum* L.) based on their susceptibility or resistance to *Z. tritici*, as described in the Swiss granum website (https://www.swissgranum.ch/documents/741931/1152834/LES_Winterweizen_2020.pdf/e624760c-8329-e11d-7e53-afbec9146156). Runal and Claro are classified as susceptible cultivars. Arina is considered intermediate, whereas Titlis and Camedo are described as resistant cultivars to *Z. tritici.* On the left panel, *ΔZtKu70* and *ΔZtSof1* showed comparable host damage for the Runal, Claro, and Titlis cultivars at 14 dpi. Both strains were avirulent in Arina and Camedo. On the right panel, the asexual reproductive structures were observed within the necrosis of those plants inoculated with the *ΔZtKu70* strain after 21 days of infection*.* No pycnidium was observed for plants sprayed with the *ΔZtSof1* mutant, demonstrating that the failure to undergo asexual reproduction is associated with the disruption of *ZtSof1* *per se* than a cultivar-specific interaction*.*

**Additional file 12: Figure S12.** Confocal microscopy images and schematic demonstration of hyphal penetration, substomatal colonization, and initial stages of pycnidial development. Susceptible wheat cultivar Drifter was inoculated with the fluorescent 1E4_GFP_ (wild-type) and *ΔZtSof1*_GFP_ strains and monitored by confocal microscopy at different days post-infection (dpi). At 6 dpi, the epiphytic filamentous hyphae penetrate the host tissue through stomatal openings. At 7 dpi, the fungus initiated the intracellular hyphal colonization of the substomatal chamber. The filamentous surrounding the stomatal guard cells produce specialized knots from where secondary hyphae emerge and germinate. Up to this point, none morphological difference of hyphal extension or intracellular hyphal colonization was noticed between 1E4_GFP_ and *ΔZtSof1*_GFP_ strains. At 8 dpi, the secondary hyphae fuse with another nearby hypha (represented by black circles) in the 1E4_GFP_ strain, creating an interconnected network in the sub-stomatal cavity. Unlike, the secondary hyphae of the *ΔZtSof1*_GFP_ mutant kept extending as individual filamentous. No anastomosis was observed until this developmental stage. For later time points (9 and 12 dpi), please see Figure 6.

**Additional file 13: Figure S13.** Description of functional characterizations performed in this study. (A) To increase the homologous recombination efficiency, we first inactivated the *ZtKu70* (*Mycgr3G85040* or *Zt09_3_00215*) gene via homologous recombination in the 1E4 wild-type (WT) strain using the plasmid pGEN-YR-*ΔZtKu70* [80], containing a geneticin resistance gene cassette (also known as G418), as a selectable marker. To disrupt the *Z. tritici So* gene (*ZtSof1*), 1 Kb size of both flanking regions were amplified from the 1E4-WT genomic DNA. The hygromycin resistance gene cassette (*hph*), used as a selective marker, was amplified from pES6 plasmid (obtained from E. H. Stukenbrock, Kiel University, unpublished). The pES1 plasmid (obtained from E. H. Stukenbrock, Kiel University, unpublished) was digested with *Kpn*I and *Sbf*I for plasmid linearization, and three fragments were assembled, which resulted in the pES1-*ΔZtSof1.* The *ZtSof1* gene was knocked-out via homologous recombination in the genetic background of the 1E4*ΔZtKu70* mutant, generating the double mutant 1E4*ΔZtKu70ΔZtSof1*. To reintroduce the *ZtSof1* gene into the *ΔZtSof1* mutant strain, we used the plasmid pES1. We amplified the nourseothricin resistance gene cassette (*nat*) from pES43 plasmid (obtained from E. H. Stukenbrock, Kiel University, unpublished) to be used as a selectable marker. *ZtSof1* gene containing 1 Kb size of each flank region and the *nat* resistance gene cassette were assembled into pES1, resulting in pES1-*ΔZtSof1*-comp that allowed to introduce the *ZtSof1* gene into its native location, generating the 1E4*ΔZtKu70ΔZtSof1*-comp mutant. (B) Agarose gel shows the PCR fragments at the expected sizes of 3’954 base pairs (bp) or 1’626 bp, confirming the presence of the *ZtSof1* native gene or the hygromycin resistance gene, respectively. (C) Agarose gel shows a PCR fragment of 286 bp, confirming the presence of the nourseothricin resistance gene only in the 1E4*ΔZtKu70ΔZtSof1*-comp mutant.

**Additional file 14: Table S1.** List of primers used in this study for cloning, sequencing, and mutant confirmation.

## Data Availability

All data generated or analyzed during this study are included in this published article and its supplementary information files.

## Abbreviations

VHF: Vegetative hyphal fusion
CAT: Conidial anastomosis tube
MAPK: Mitogen-activated protein kinase
CWI: Cell wall integrity
ZtKu70: *Zymoseptoria tritici* heterodimer regulator of the NHEJ pathway
ZtSof1: *Zymoseptoria tritici* soft gene

## References

[1] Endler JA (1993). Some general comments on the evolution and design of animal communication systems. Philos Trans R Soc B.

[2] Gillam E (2011). An introduction to animal communication. Nat Educ Knowl.

[3] Wilson EO. Sociobiology: the new synthesis. United States: Harvard University Press; 1975. https://www.hup.harvard.edu/catalog.php?isbn=9780674002357.

[4] van Gestel J, Nowak MA, Tarnita CE (2012). The evolution of cell-to-cell communication in a sporulating bacterium. PLoS Comput Biol.

[5] Shrout JD, Tolker-Nielsen T, Givskov M, Parsek MR (2011). The contribution of cell-cell signaling and motility to bacterial biofilm formation. MRS Bull.

[6] Fischer MS, Glass NL (2019). Communicate and fuse: how filamentous fungi establish and maintain an interconnected mycelial network. Front Microbiol.

[7] Cottier F, Muhlschlegel FA (2012). Communication in fungi. Int J Microbiol.

[8] Bloemendal S, Kuck U (2013). Cell-to-cell communication in plants, animals, and fungi: a comparative review. Naturwissenschaften.

[9] Wongsuk T, Pumeesat P, Luplertlop N (2016). Fungal quorum sensing molecules: role in fungal morphogenesis and pathogenicity. J Basic Microbiol.

[10] Glass NL, Jacobson DJ, Shiu PKT (2000). The genetics of hyphal fusion and vegetative incompatibility in filamentous ascomycete fungi. Annu Rev Genet.

[11] Fleissner A, Leeder AC, Roca MG, Read ND, Glass NL (2009). Oscillatory recruitment of signaling proteins to cell tips promotes coordinated behavior during cell fusion. Proc Natl Acad Sci U S A.

[12] Saupe SJ (2000). Molecular genetics of heterokaryon incompatibility in filamentous ascomycetes. Microbiol Mol Biol Rev.

[13] Read ND, Fleissner A, Roca MG, Glass NL, Borkovich KA, Edolle D (2010). Hyphal fusion. Cellular and molecular biology of filamentous fungi.

[14] Hickey PC, Jacobson DJ, Read ND, Glass NL (2002). Live-cell imaging of vegetative hyphal fusion in Neurospora crassa. Fungal Genet Biol.

[15] Craven KD, Velez H, Cho Y, Lawrence CB, Mitchell TK (2008). Anastomosis is required for virulence of the fungal necrotroph Alternaria brassicicola. Eukaryot Cell.

[16] Prados Rosales RC, Di Pietro A (2008). Vegetative hyphal fusion is not essential for plant infection by Fusarium oxysporum. Eukaryot Cell.

[17] Charlton ND, Shoji JY, Ghimire SR, Nakashima J, Craven KD (2012). Deletion of the fungal gene soft disrupts mutualistic symbiosis between the grass endophyte Epichloe festucae and the host plant. Eukaryot Cell.

[18] Roca GM, Read ND, Wheals AE (2005). Conidial anastomosis tubes in filamentous fungi. FEMS Microbiol Lett.

[19] Mehrabi R, Bahkali AH, Abd-Elsalam KA, Moslem M, Ben M’barek S, Gohari AM, Jashni MK, Stergiopoulos I, Kema GH, de Wit PJ (2011). Horizontal gene and chromosome transfer in plant pathogenic fungi affecting host range. FEMS Microbiol Rev.

[20] Friesen TL, Stukenbrock EH, Liu Z, Meinhardt S, Ling H, Faris JD, Rasmussen JB, Solomon PS, McDonald BA, Oliver RP (2006). Emergence of a new disease as a result of interspecific virulence gene transfer. Nat Genet.

[21] Temporini ED, VanEtten HD (2004). An analysis of the phylogenetic distribution of the pea pathogenicity genes of Nectria haematococca MPVI supports the hypothesis of their origin by horizontal transfer and uncovers a potentially new pathogen of garden pea: Neocosmospora boniensis. Curr Genet.

[22] Roca MG, Davide LC, Davide LM, Mendes-Costa MC, Schwan RF, Wheals AE (2004). Conidial anastomosis fusion between Colletotrichum species. Mycol Res.

[23] He C, Rusu AG, Poplawski AM, Irwin JAG, Manners JM (1998). Transfer of a supernumerary chromosome between vegetatively incompatible biotypes of the fungus Colletotrichum gloeosporioides. Genet Soc Am.

[24] Fleissner A, Sarkar S, Jacobson DJ, Roca MG, Read ND, Glass NL (2005). The so locus is required for vegetative cell fusion and postfertilization events in Neurospora crassa. Eukaryot Cell.

[25] Teichert I, Steffens EK, Schnass N, Franzel B, Krisp C, Wolters DA, Kuck U (2014). PRO40 is a scaffold protein of the cell wall integrity pathway, linking the MAP kinase module to the upstream activator protein kinase C. PLoS Genet.

[26] Weichert M, Lichius A, Priegnitz BE, Brandt U, Gottschalk J, Nawrath T, Groenhagen U, Read ND, Schulz S, Fleissner A (2016). Accumulation of specific sterol precursors targets a MAP kinase cascade mediating cell-cell recognition and fusion. Proc Natl Acad Sci U S A.

[27] Yin Y, Wu S, Chui C, Ma T, Jiang H, Hahn M, Ma Z (2018). The MAPK kinase BcMkk1 suppresses oxalic acid biosynthesis via impeding phosphorylation of BcRim15 by BcSch9 in Botrytis cinerea. PLoS Pathog.

[28] Fleissner A, Glass NL (2007). SO, a protein involved in hyphal fusion in Neurospora crassa, localizes to septal plugs. Eukaryot Cell.

[29] Maruyama J, Escano CS, Kitamoto K (2010). AoSO protein accumulates at the septal pore in response to various stresses in the filamentous fungus Aspergillus oryzae. Biochem Biophys Res Commun.

[30] Engh I, Wurtz C, Witzel-Schlomp K, Zhang HY, Hoff B, Nowrousian M, Rottensteiner H, Kuck U (2007). The WW domain protein PRO40 is required for fungal fertility and associates with Woronin bodies. Eukaryot Cell.

[31] Fones H, Gurr S (2015). The impact of Septoria tritici blotch disease on wheat: an EU perspective. Fungal Genet Biol.

[32] Motteram J, Lovegrove A, Pirie E, Marsh J, Devonshire J, van de Meene A, Hammond-Kosack K, Rudd JJ (2011). Aberrant protein N-glycosylation impacts upon infection-related growth transitions of the haploid plant-pathogenic fungus Mycosphaerella graminicola. Mol Microbiol.

[33] Mehrabi R, Zwiers LH, de Waard MA, Kema GH (2006). MgHog1 regulates dimorphism and pathogenicity in the fungal wheat pathogen Mycosphaerella graminicola. Mol Plant-Microbe Interact.

[34] Francisco CS, Ma X, Zwyssig MM, McDonald BA, Palma-Guerrero J (2019). Morphological changes in response to environmental stresses in the fungal plant pathogen Zymoseptoria tritici. Sci Rep.

[35] Shipton WA, Boyd WRJ, Rosielle AA, Shearer BI (1971). The common Septoria diseases of wheat. Bot Rev.

[36] Palma-Guerrero J, Ma X, Torriani SF, Zala M, Francisco CS, Hartmann FE, Croll D, McDonald BA (2017). Comparative transcriptome analyses in Zymoseptoria tritici reveal significant differences in gene expression among strains during plant infection. Mol Plant-Microbe Interact.

[37] Karisto P, Hund A, Yu K, Anderegg J, Walter A, Mascher F, McDonald BA, Mikaberidze A (2018). Ranking quantitative resistance to Septoria tritici blotch in elite wheat cultivars using automated image analysis. Phytopathology.

[38] Dean R, Van Kan JA, Pretorius ZA, Hammond-Kosack KE, Di Pietro A, Spanu PD, Rudd JJ, Dickman M, Kahmann R, Ellis J (2012). The top 10 fungal pathogens in molecular plant pathology. Mol Plant Pathol.

[39] Mehrabi R, Ben M’Barek S, van der Lee TA, Waalwijk C, de Wit PJ, Kema GH (2009). G(alpha) and Gbeta proteins regulate the cyclic AMP pathway that is required for development and pathogenicity of the phytopathogen Mycosphaerella graminicola. Eukaryot Cell.

[40] Gohari AM, Mehrabi R, Robert O, Ince IA, Boeren S, Schuster M, Steinberg G, de Wit PJ, Kema GH (2014). Molecular characterization and functional analyses of ZtWor1, a transcriptional regulator of the fungal wheat pathogen Zymoseptoria tritici. Mol Plant Pathol.

[41] Kilaru S, Schuster M, Ma W, Steinberg G (2017). Fluorescent markers of various organelles in the wheat pathogen Zymoseptoria tritici. Fungal Genet Biol.

[42] Simonin A, Palma-Guerrero J, Fricker M, Glass NL (2012). Physiological significance of network organization in fungi. Eukaryot Cell.

[43] Roca MG, Arlt J, Jeffree CE, Read ND (2005). Cell biology of conidial anastomosis tubes in Neurospora crassa. Eukaryot Cell.

[44] Vitale S, Di Pietro A, Turra D (2019). Autocrine pheromone signalling regulates community behaviour in the fungal pathogen Fusarium oxysporum. Nat Microbiol.

[45] Ishikawa FH, Souza EA, Read ND, Roca MG (2010). Live-cell imaging of conidial fusion in the bean pathogen, Colletotrichum lindemuthianum. Fungal Biol.

[46] Chagnon PL (2014). Ecological and evolutionary implications of hyphal anastomosis in arbuscular mycorrhizal fungi. FEMS Microbiol Ecol.

[47] Roper M, Ellison C, Taylor JW, Glass NL (2011). Nuclear and genome dynamics in multinucleate ascomycete fungi. Curr Biol.

[48] Biella S, Smith ML, Aist JR, Cortesi P, Milgroom MG (2002). Programmed cell death correlates with virus transmission in a filamentous fungus. Proc Biol Sci.

[49] Goddard MR, Burt A (1999). Recurrent invasion and extinction of a selfish gene. PNAS.

[50] Deng YZ, Zhang B, Chang C, Wang Y, Lu S, Sun S, Zhang X, Chen B, Jiang Z (2018). The MAP kinase SsKpp2 is required for mating/filamentation in Sporisorium scitamineum. Front Microbiol.

[51] Zhao X, Mehrabi R, Xu JR (2007). Mitogen-activated protein kinase pathways and fungal pathogenesis. Eukaryot Cell.

[52] Pandey A, Roca MG, Read ND, Glass NL (2004). Role of a mitogen-activated protein kinase pathway during conidial germination and hyphal fusion in Neurospora crassa. Eukaryot Cell.

[53] Maddi A, Dettman A, Fu C, Seiler S, Free SJ (2012). WSC-1 and HAM-7 are MAK-1 MAP kinase pathway sensors required for cell wall integrity and hyphal fusion in Neurospora crassa. PLoS One.

[54] Leng Y, Zhong S (2015). The role of mitogen-activated protein (MAP) kinase signaling components in the fungal development, stress response and virulence of the fungal cereal pathogen Bipolaris sorokiniana. PLoS One.

[55] Hagiwara D, Sakamoto K, Abe K, Gomi K (2016). Signaling pathways for stress responses and adaptation in Aspergillus species: stress biology in the post-genomic era. Biosci Biotechnol Biochem.

[56] Mehrabi R, van der Lee T, Waalwijk C, Kema GH (2006). MgSlt2, a cellular integrity MAP kinase gene of the fungal wheat pathogen Mycosphaerella graminicola, is dispensable for penetration but essential for invasive growth. Mol Plant-Microbe Interact.

[57] Cousin A, Mehrabi R, Guilleroux M, Dufresne M, VAN DER Lee T, Waalwijk C, Langin T, Kema GH (2006). The MAP kinase-encoding gene MgFus3 of the non-appressorium phytopathogen Mycosphaerella graminicola is required for penetration and in vitro pycnidia formation. Mol Plant Pathol.

[58] Read ND, Lichius A, Shoji JY, Goryachev AB (2009). Self-signalling and self-fusion in filamentous fungi. Curr Opin Microbiol.

[59] Krishnan P, Meile L, Plissonneau C, Ma X, Hartmann FE, Croll D, McDonald BA, Sanchez-Vallet A (2018). Transposable element insertions shape gene regulation and melanin production in a fungal pathogen of wheat. BMC Biol.

[60] Lendenmann MH, Croll D, Stewart EL, McDonald BA (2014). Quantitative trait locus mapping of melanization in the plant pathogenic fungus Zymoseptoria tritici. G3 (Bethesda).

[61] Yago JI, Lin CH, Chung KR (2011). The SLT2 mitogen-activated protein kinase-mediated signalling pathway governs conidiation, morphogenesis, fungal virulence and production of toxin and melanin in the tangerine pathotype of Alternaria alternata. Mol Plant Pathol.

[62] Liu W, Soulie MC, Perrino C, Fillinger S (2011). The osmosensing signal transduction pathway from Botrytis cinerea regulates cell wall integrity and MAP kinase pathways control melanin biosynthesis with influence of light. Fungal Genet Biol.

[63] Valiante V, Macheleidt J, Foge M, Brakhage AA (2015). The Aspergillus fumigatus cell wall integrity signaling pathway: drug target, compensatory pathways, and virulence. Front Microbiol.

[64] Wei W, Xiong Y, Zhu W, Wang N, Yang G, Peng F (2016). Colletotrichum higginsianum mitogen-activated protein kinase ChMK1: role in growth, cell wall integrity, colony melanization, and pathogenicity. Front Microbiol.

[65] Manfiolli AO, Siqueira FS, Dos Reis TF, Van Dijck P, Schrevens S, Hoefgen S, Foge M, Strassburger M, de Assis LJ, Heinekamp T, et al. Mitogen-activated protein kinase cross-talk interaction modulates the production of melanins in Aspergillus fumigatus. mBio. 2019;10(2).. 10.1128/mBio.00215-19 PMC6437049 30914505

[66] Song Z, Zhong Q, Yin Y, Shen L, Li Y, Wang Z (2016). The high osmotic response and cell wall integrity pathways cooperate to regulate morphology, microsclerotia development, and virulence in Metarhizium rileyi. Sci Rep.

[67] Valiante V. The cell wall integrity signaling pathway and its involvement in secondary metabolite production. J Fungi (Basel). 2017;3(4). 10.3390/jof3040068 PMC5753170 29371582

[68] Tiley AMM, Foster GD, Bailey AM (2018). Exploring the genetic regulation of asexual sporulation in Zymoseptoria tritici. Front Microbiol.

[69] Mehrabi R, Kema GH (2006). Protein kinase A subunits of the ascomycete pathogen Mycosphaerella graminicola regulate asexual fructification, filamentation, melanization and osmosensing. Mol Plant Pathol.

[70] Duncan KE, Howard RJ (2000). Cytological analysis of wheat infection by the leaf blotch pathogen Mycosphaerella graminicola. Mycol Res.

[71] Zhan J, Kema GH, Waalwijk C, McDonald BA (2002). Distribution of mating type alleles in the wheat pathogen Mycosphaerella graminicola over spatial scales from lesions to continents. Fungal Genet Biol.

[72] Kema GH, van Silfhout CH (1997). Genetic variation for virulence and resistance in the wheat-*Mycosphaerella graminicola* pathosystem III. Comparative seedling and adult plant experiments. Phytopathology.

[73] Meile L, Croll D, Brunner PC, Plissonneau C, Hartmann FE, McDonald BA, Sanchez-Vallet A. A fungal avirulence factor encoded in a highly plastic genomic region triggers partial resistance to septoria tritici blotch. New Phytol. 2018;219:1048–61. 10.1111/nph.15180 PMC6055703 29693722

[74] Schneider CA, Rasband WS, Eliceiri KW (2012). NIH Image to ImageJ: 25 years of image analysis. Nat Methods.

[75] Wickham H (2009). ggplot2: elegant graphics for data analysis.

[76] Mendiburu FD (2015). Agricolae: statistical procedures for agricultural research. R Package Version 1.2-3.

[77] Stewart EL, Hagerty CH, Mikaberidze A, Mundt CC, Zhong Z, McDonald BA (2016). An improved method for measuring quantitative resistance to the wheat pathogen Zymoseptoria tritici using high-throughput automated image analysis. Phytopathology.

[78] Larsson A (2014). AliView: a fast and lightweight alignment viewer and editor for large datasets. Bioinformatics.

[79] Tamura K, Stecher G, Peterson D, Filipski A, Kumar S (2013). MEGA6: molecular evolutionary genetics analysis version 6.0. Mol Biol Evol.

[80] Sidhu YS, Cairns TC, Chaudhari YK, Usher J, Talbot NJ, Studholme DJ, Csukai M, Haynes K (2015). Exploitation of sulfonylurea resistance marker and non-homologous end joining mutants for functional analysis in Zymoseptoria tritici. Fungal Genet Biol.

