# Supplementary figures and images for: The role of vegetative cell fusions in the development and asexual reproduction of the wheat fungal pathogen Zymoseptoria tritici
Source: BMC Biol. 2020 Aug 11;18:99. doi: 10.1186/s12915-020-00838-9 (PMC7477884; doi:10.1186/s12915-020-00838-9)

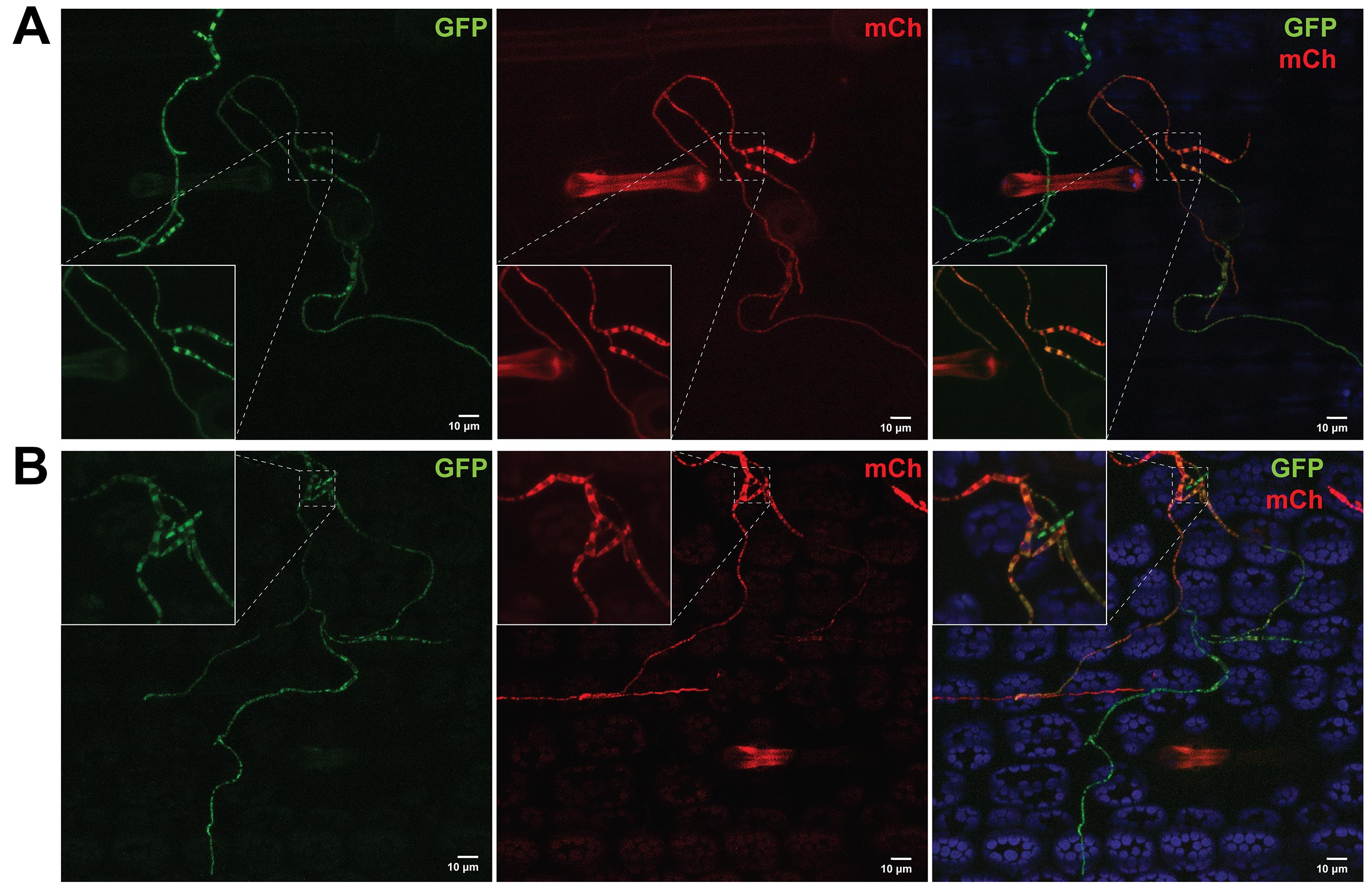

Supplement: Supplementary file 1 — Additional file 1: Figure S1. Vegetative hyphal fusion occurs during epiphytic growth on wheat leaves. Co-infection of wheat plants with blastospores (A) or pycnidiospores (B) from the 1E4 strain expressing either the cytoplasmic green fluorescent protein (GFP) or the red-fluorescent protein (mCherry) resulted in hyphal fusions and cytoplasmic exchange after 48 hours of infection. Hyphal fusion during epiphytic colonization may assist the fungus to create an interconnected network supporting its establishment on the leaf surface before host penetration. [file 12915_2020_838_MOESM1_ESM.jpg]

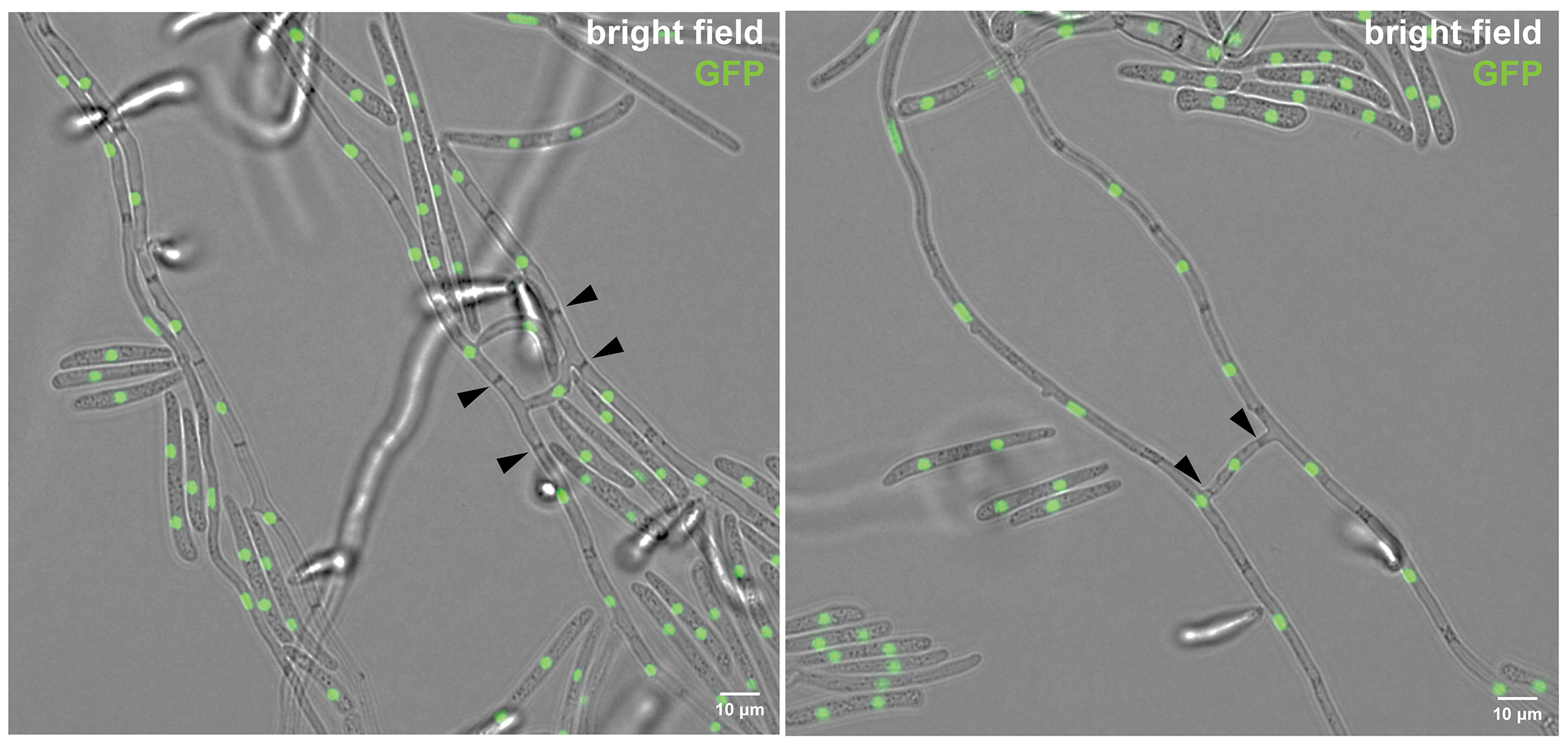

Supplement: Supplementary file 2 — Additional file 2: Figure S2. Hyphal fusion does not lead to the generation of multinucleated cells in Zymoseptoria tritici. Blastospores of the IPO323 ZtHis1-ZtGFP strain, which has the GFP as a fluorescent marker labeling the nucleus, were plated on WA plates at a final concentration of 106 blastospores/mL and incubated at 18°C. After 72 hours of incubation, none septum containing more than one nucleus was observed neither at hyphal bridges nor distant of the fusion point. Black triangles indicate the septal compartment containing only one nucleus at the fusion bridges. [file 12915_2020_838_MOESM2_ESM.jpg]

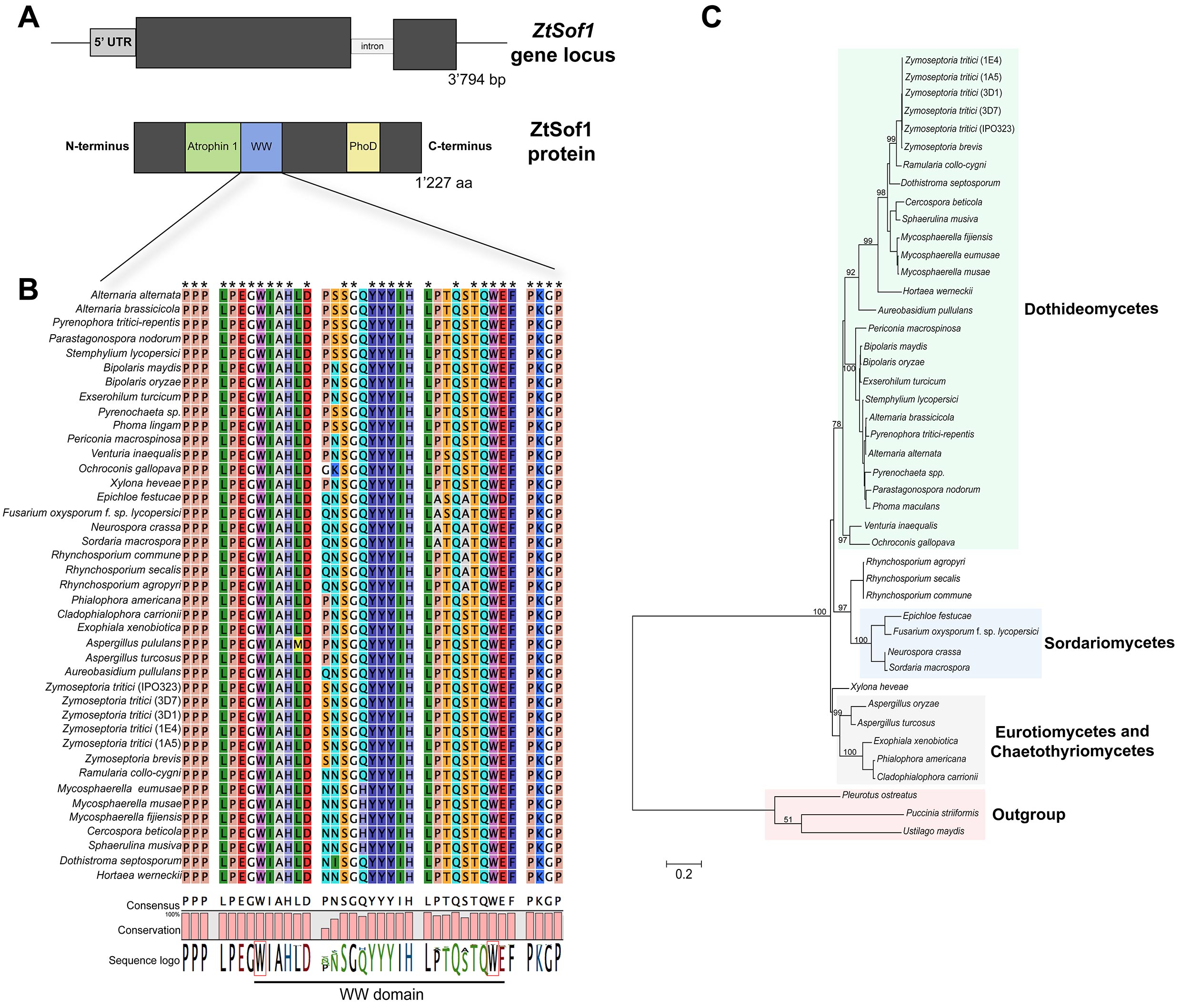

Supplement: Supplementary file 3 — Additional file 3: Figure S3. Scheme showing the phylogenetic relationship of the So gene within Ascomycete species. The so gene sequence from Neurospora crassa (XM_958983.3) was blasted against the Z. tritici genome (https://genome.jgi.doe.gov/Mycgr3/Mycgr3.home.html) to identify its orthologous in this fungus. The Z. tritici So orthologous protein sequence was used for a Blastp analysis against the NCBI database (National Center of Biotechnology Information). Blastp searches at expected value homology cut-off of 1e-10 were included as positive. A dataset containing So orthologous proteins of different Ascomycete species were used for phylogenetic analysis. Protein sequences were aligned using the AliView program [78]. The best-fit model of amino acid evolution was the LG+G, determined by Mega6 software [79]. Amino acid sequences were aligned using Muscle, followed by maximum likelihood (ML) phylogeny reconstruction using 1,000 bootstraps and performed with the software Mega6 [79]. (A) The illustration demonstrates the ZtSof1 gene locus and its protein sequence containing the Atrophin 1, WW, and PhoD, as protein domains. Comparison of ZtSof1 protein sequence with its orthologs showed 53% identity with Epichloe festucae; 54% identity with Neurospora crassa and Sordaria macrospora; 55% identity with Fusarium oxysporum; 60% identity with Aspergillus oryzae; and 63% identity with Alternaria brassicicola. (B) The alignment of the WW protein-protein interaction domain, including the PPLP motif of 41 different fungal species. Red boxes surround the two conserved tryptophan residues spaced by 22 amino acids apart. (C) Phylogenetic analysis grouped the orthologs of the ZtSof1 gene onto three groups based on fungal Classes (Dothideomycetes, Sordariomycetes, and Chaetothyriomycetes together with Eurotiomycetes), independently whether they were parasites, mutualists or saprotrophs. Three members of Basidiomycetes were used as an outgroup to root the tree. [file 12915_2020_838_MOESM3_ESM.jpg]

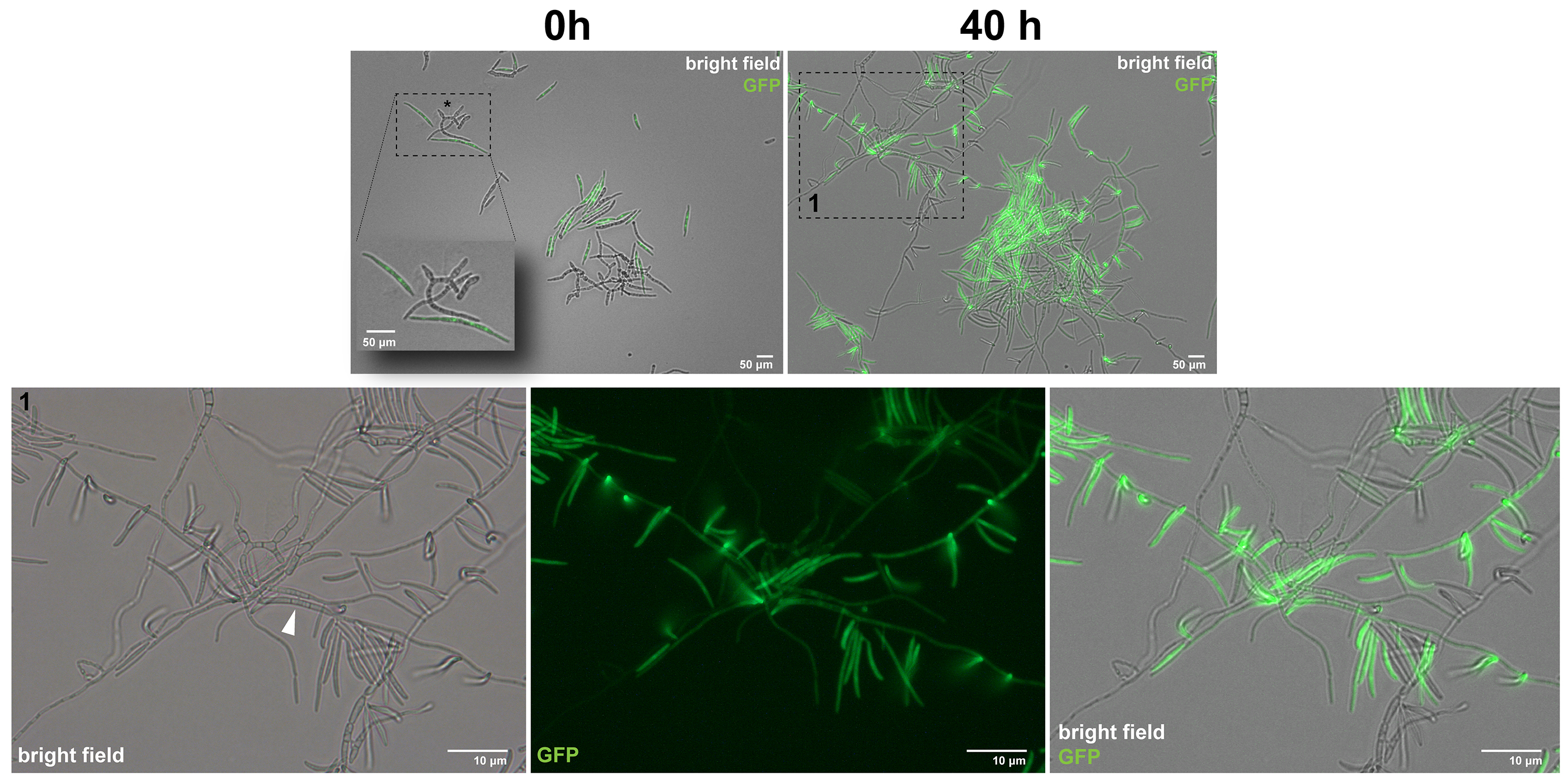

Supplement: Supplementary file 4 — Additional file 4: Figure S4. Cytoplasmic streaming between ΔZtKu70 and the GFP-tagged 1E4 strain. Blastospores of ΔZtKu70 and 1E4GFP were co-inoculated on water agar (WA) plates, a hyphal fusion-inducing condition. After 40 hours of incubation, fusion bridges were observed between ΔZtKu70 and 1E4GFP strains. The detection of the green fluorescent protein in the cytoplasm of the recipient hypha ΔZtKu70 confirms the cytoplasmic streaming between the two fused individuals (panel 1). Black asterisk points to the non-fluorescent ΔZtKu70 spore before hyphal fusion. White triangle indicates the fusion point between the ΔZtKu70 and 1E4GFP strains. [file 12915_2020_838_MOESM4_ESM.jpg]

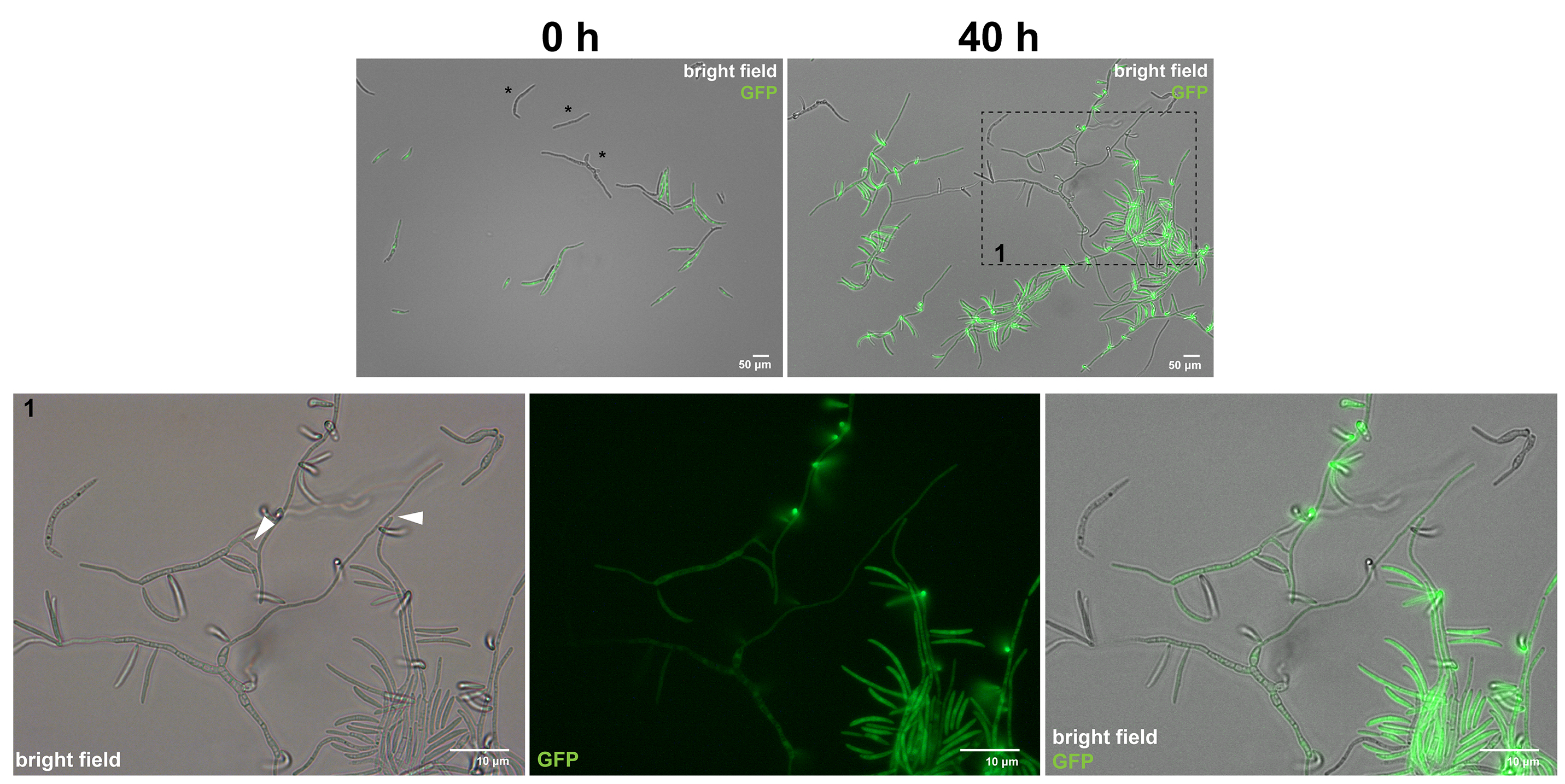

Supplement: Supplementary file 5 — Additional file 5: Figure S5. Cytoplasmic streaming between ΔZtSof1-comp and GFP-tagged 1E4 strain. Blastospores of ΔZtSof1-comp and 1E4GFP were co-inoculated on water agar (WA) plates, a hyphal fusion-inducing condition. After 40 hours of incubation, fusion bridges were observed between ΔZtSof1-comp and 1E4GFP strains. The detection of the green fluorescent protein in the cytoplasm of the recipient hyphae ΔZtSof1-comp confirms the cytoplasmic streaming between the fused individuals (panel 1). Black asterisks point to the non-fluorescent ΔZtSof1-comp spore before hyphal fusion. White triangles indicate the fusion points between the ΔZtSof1-comp and 1E4(GFP) strains. [file 12915_2020_838_MOESM5_ESM.jpg]

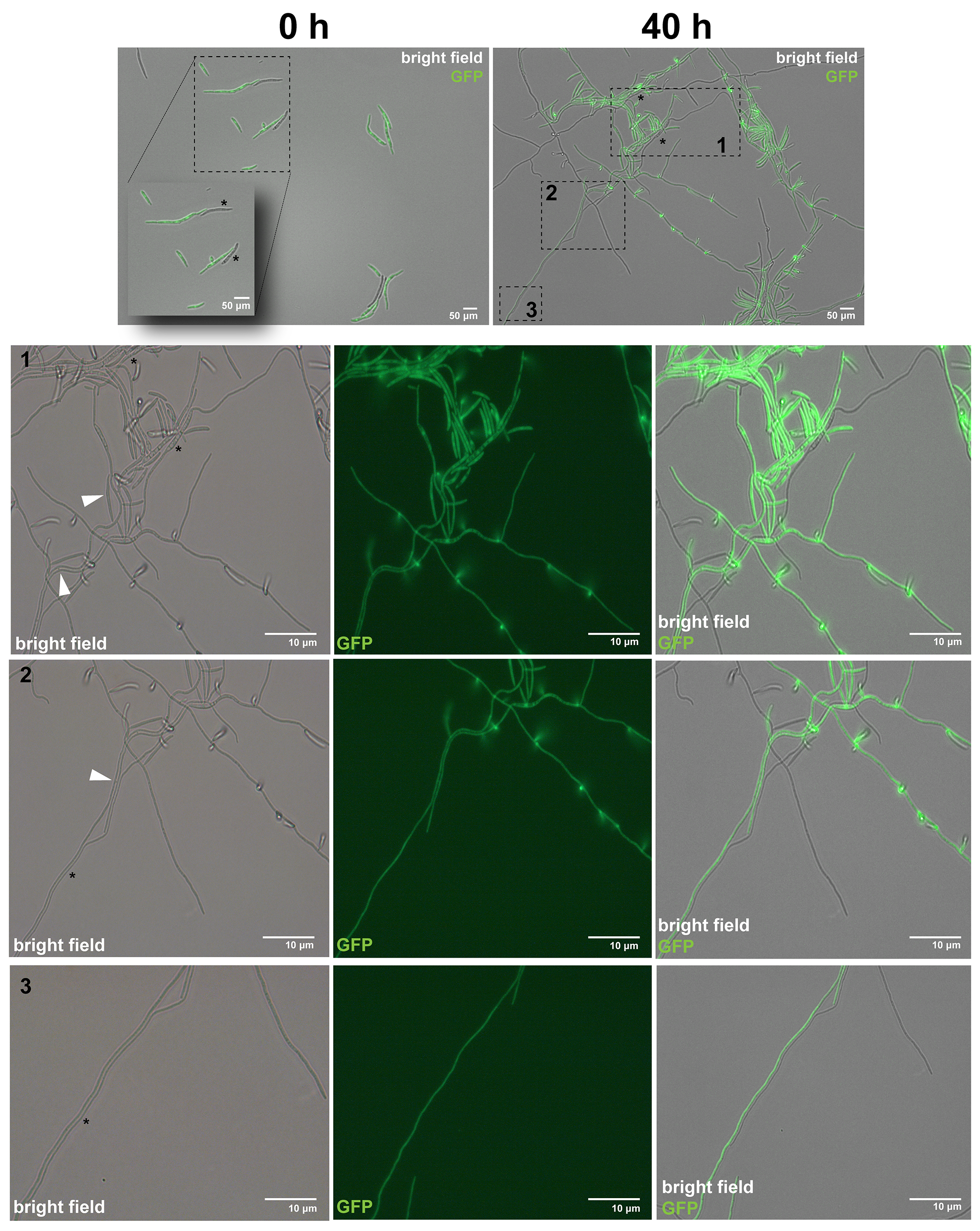

Supplement: Supplementary file 6 — Additional file 6: Figure S6. Co-inoculation of ΔZtSof1 and GFP-tagged 1E4 strain confirms the failure of the ΔZtSof1 mutant to undergo hyphal fusion. Blastospores of ΔZtSof1 and 1E4GFP were co-inoculated on water agar (WA) plates, a hyphal fusion-inducing condition. After 40 hours of incubation, fusion bridges were only observed between 1E4GFP germinating spores (panel 1). Fluorescent green protein was never detected on the cytoplasm of ΔZtSof1 cells (panels 1, 2, and 3). The filamentous of the ΔZtSof1 mutant grew in parallel with those hyphae from the 1E4GFP, but they never undergo hyphal fusion (panels 2 and 3), demonstrating that ZtSof1 is required in both fusion partners to establish the fungal communication required for perception or response during cell fusion. Black asterisks point to the non-fluorescent ΔZtSof1 spores. [file 12915_2020_838_MOESM6_ESM.jpg]

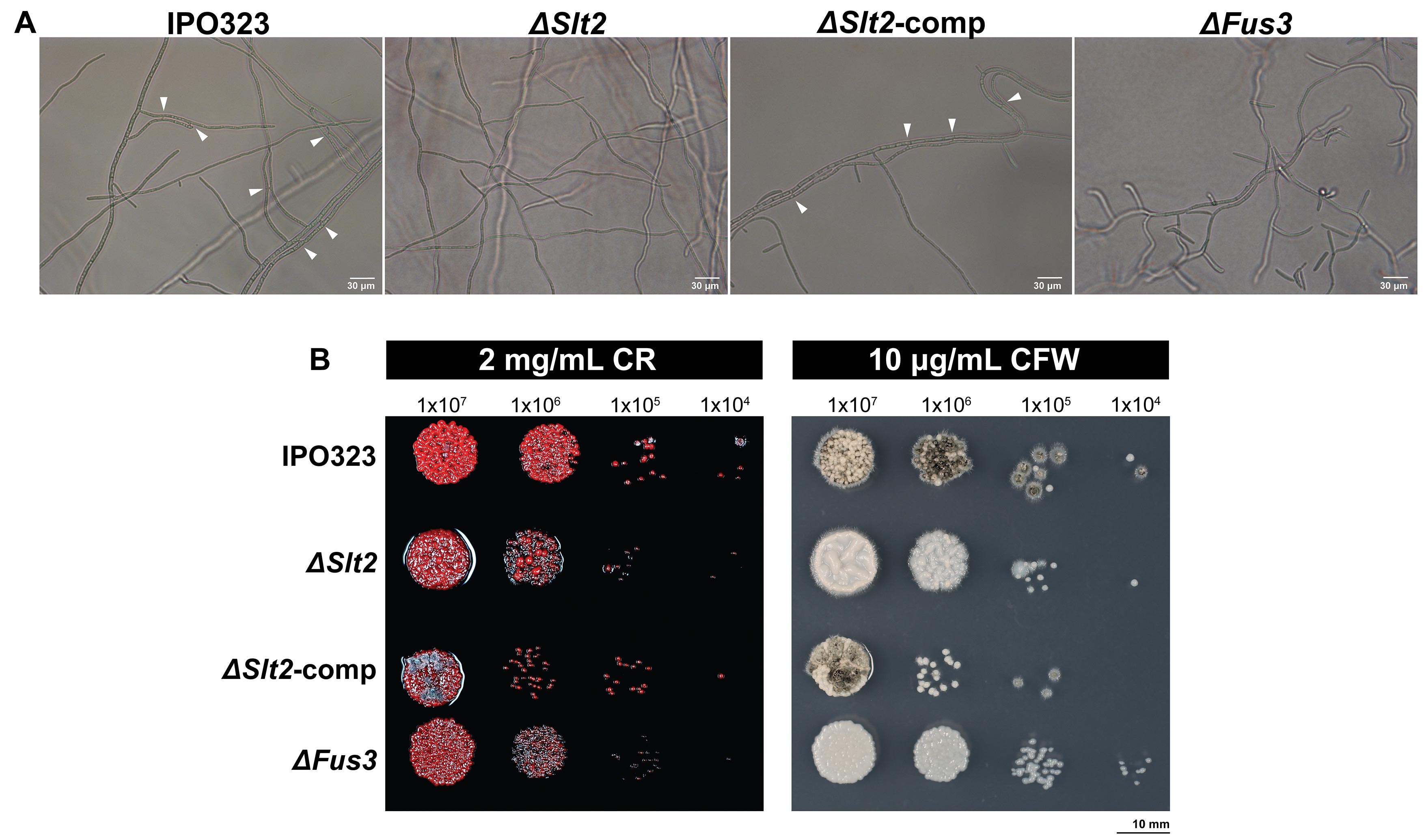

Supplement: Supplementary file 7 — Additional file 7: Figure S7. MAPK-encoding ZtSlt2 and ZtFus3 genes are required for anastomosis in Zymoseptoria tritici. (A) Hyphal fusions were regularly found in the wild-type strain (IPO323). The deletion of ZtSlt2 (orthologous to MAK-1) or ZtFus3 (orthologous to MAK-2) resulted in fusion-defective mutants, probably due to the disruption of the oscillatory recruitment of both MAPK modules required for cell-to-cell communication and fusion, as described for Neurospora crassa [11]. The defective phenotype was restored in the complemented ΔZtSlt2-comp strain. White triangles point to self-fusion events. (B) Deletion of the MAPK Slt2 or Fus3 is dispensable for the cellular integrity of Z. tritici. A serial dilution of blastospore suspensions of IPO323, ΔZtSlt2, ΔZtSlt2-comp, and ΔZtFus3 strains were exposed for five days to cell wall stresses (2 mg/mL Congo red - CR and 10 μg/mL Calcofluor white - CFW). The tested strains do not vary on their tolerance to the cellular stressors. [file 12915_2020_838_MOESM7_ESM.jpg]

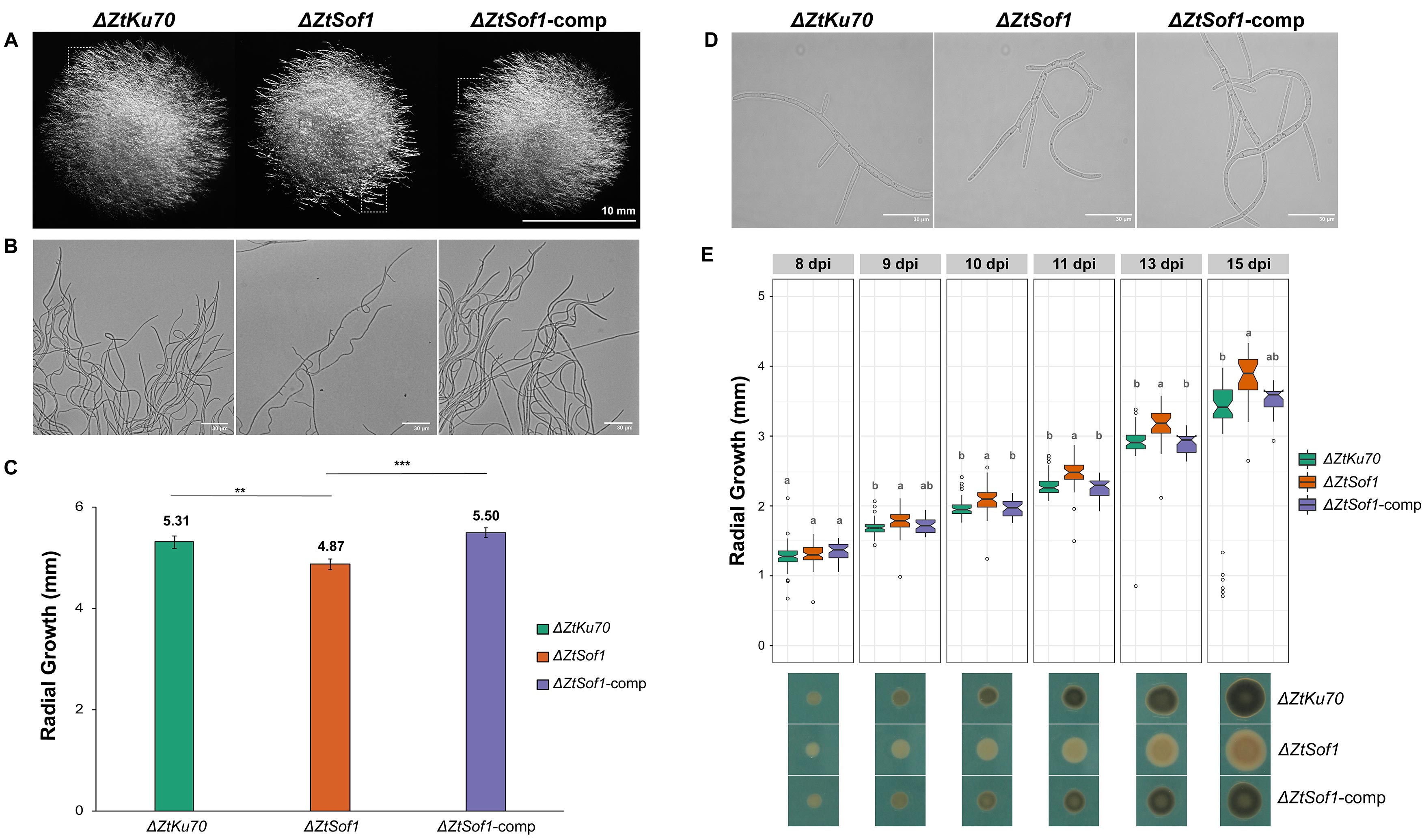

Supplement: Supplementary file 8 — Additional file 8: Figure S8. ZtSof1 impact the vegetative growth in a morphotype-depending manner in Zymoseptoria tritici. A nutrient-poor medium (WA), inducing hyphal growth, and a nutrient-rich medium (PDA), inducing blastosporulation, were used to assess the effect of ZtSof1 deletion on fungal radial growth. (A) The ΔZtSof1 mutant exhibited a similar colony morphology than ΔZtKu70 and ΔZtSof1-comp strains on WA plates. (B) Light microscopy of colony edges showed a dense hyphal-thickened margin for ΔZtKu70 and ΔZtSof1-comp colonies, whereas the ΔZtSof1 mutant exhibited only a few filamentous at the colony periphery. Dashed squares point to the localization of microscope images. (C) Thought no morphological differences were observed for the tested Z. tritici strains, the fusion defective ΔZtSof1 mutant had a slight, but significant reduction of its radial growth (mm) compared to ΔZtKu70 and ΔZtSof1-comp when grown on a nutrient-limited medium. At least 40 colonies of each tested strains were evaluated. Two and three stars indicate a p-value <0.005 and <0.0005, respectively. (D) No morphological differences were noticed for the blastospores produced by ΔZtKu70, ΔZtSof1, or ΔZtSof1-comp strains. (E) ΔZtSof1 mutant grew faster and had higher radial growth over time than the ΔZtKu70 and ΔZtSof1-comp strains when incubated on PDA. Bars represent standard errors of the radial growth (mm) of at least 40 colonies. Different letters on the top of the bars indicate a significant difference among the tested strains according to the Analysis of Variance (ANOVA). The notch displays a 95% confidence interval of the median. Open circles represent the outlier values of each strain. Pictures shown below the bar plot illustrate the colony sizes of ΔZtKu70, ΔZtSof1, and ΔZtSof1-comp strains. [file 12915_2020_838_MOESM8_ESM.jpg]

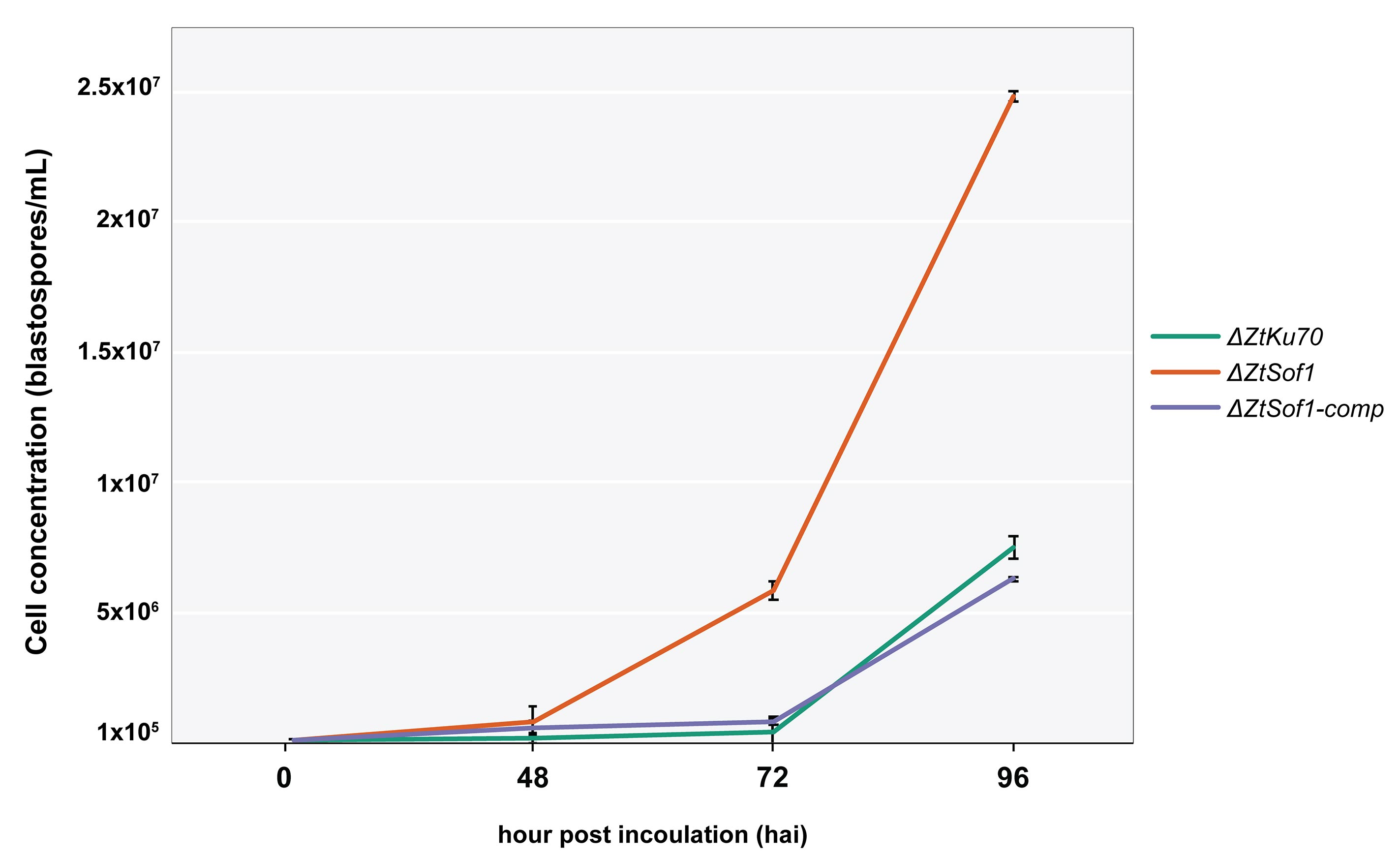

Supplement: Supplementary file 9 — Additional file 9: Figure S9. ZtSof1 deletion induced blastosporulation in Zymoseptoria tritici. The nutrient-rich YSB medium was used to access the contribution of ZtSof1 to the blastospore formation. The experiment was performed twice with similar results. [file 12915_2020_838_MOESM9_ESM.jpg]

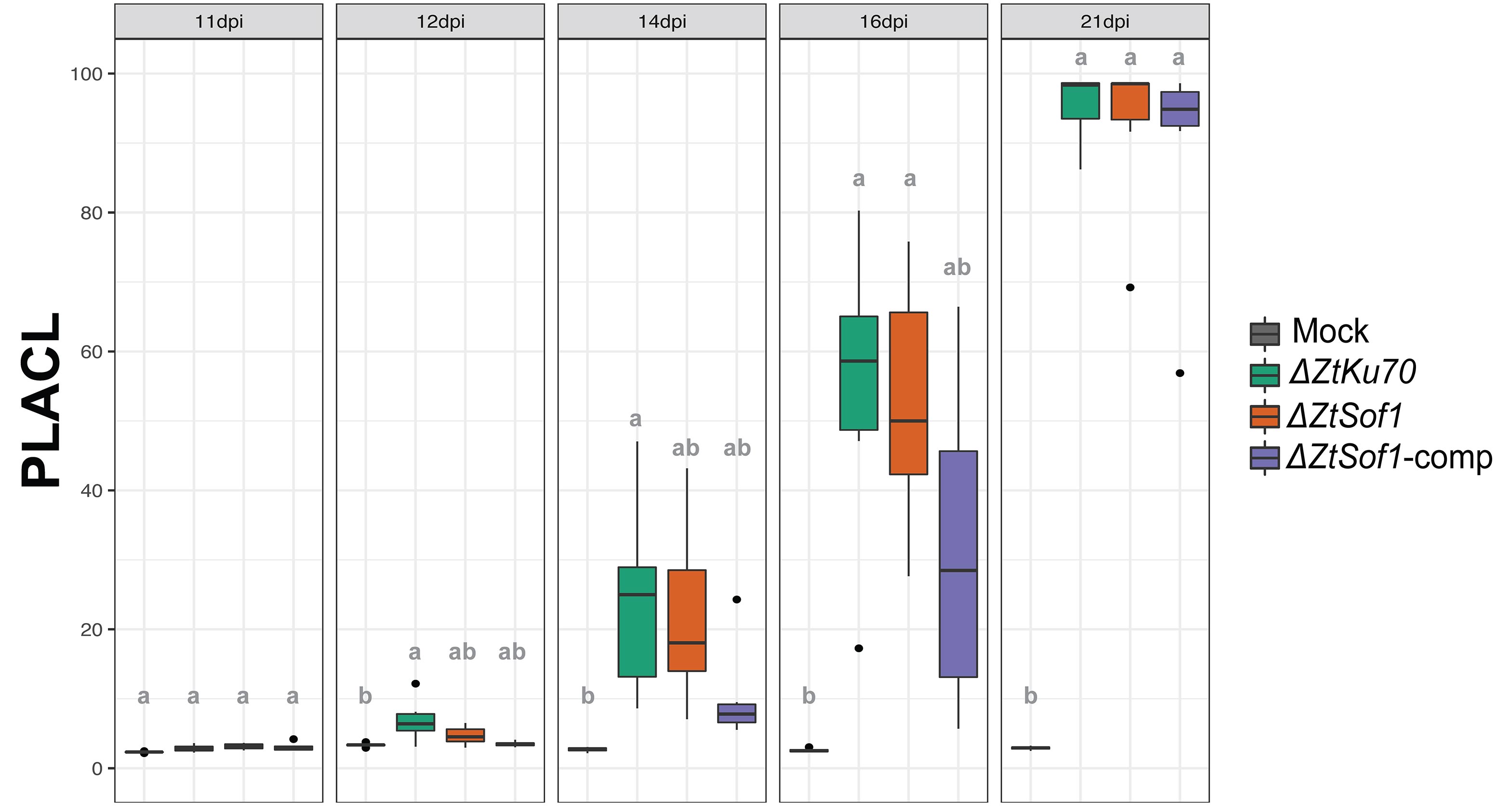

Supplement: Supplementary file 10 — Additional file 10: Figure S10. Percentage of leaves covered by lesions (PLACL). The second leaves of the wheat cultivar Drifter infected with ZtKu70, ΔZtSof1, and ΔZtSof1-comp strains and harvested at different days post-inoculation (dpi). Bars represent standard errors of PLACL values of at least six infected leaves. Different letters on the top of the bars indicate a significant difference among the tested strains according to the Analysis of Variance (ANOVA). Black circles represent the outlier data points. [file 12915_2020_838_MOESM10_ESM.jpg]

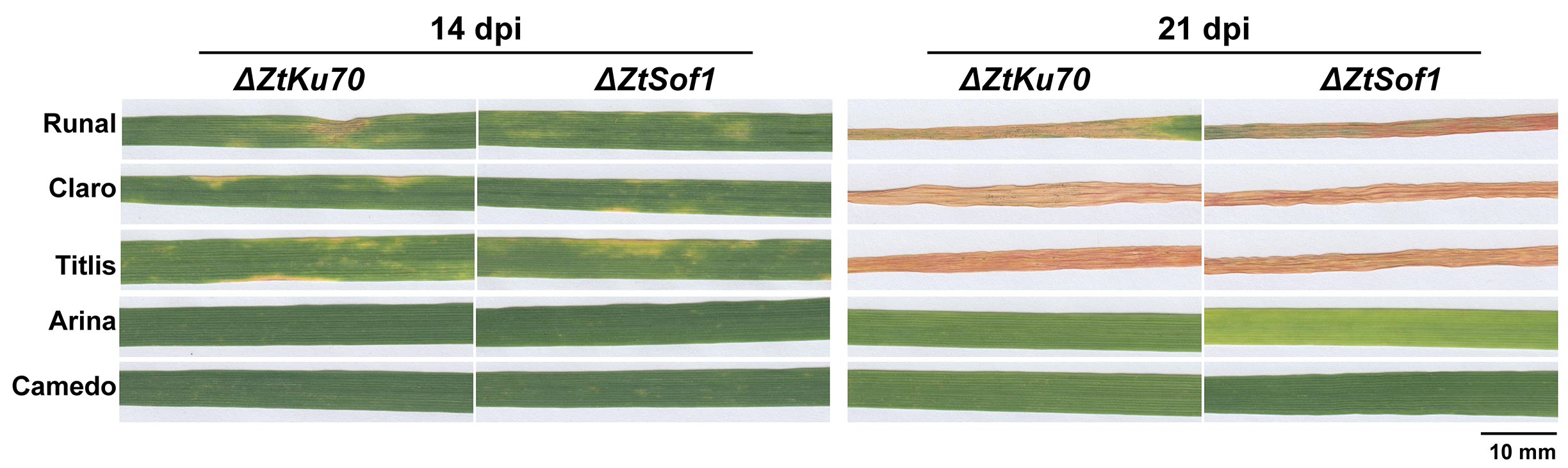

Supplement: Supplementary file 11 — Additional file 11: Figure S11. ΔZtSof1 and ΔZtKu70 strains do not vary in pathogenicity, except for the failure of the ΔZtSof1 mutant to undergo asexual reproduction. Five different winter cultivars infected with ΔZtKu70 or ΔZtSof1 strains were, respectively, evaluated at 14- and 21 days post-inoculation (dpi) for host damage and pathogen reproduction. We used five different winter cultivars of wheat (Triticum aestivum L.) based on their susceptibility or resistance to Z. tritici, as described in the Swiss granum website (https://www.swissgranum.ch/documents/741931/1152834/LES_Winterweizen_2020.pdf/e624760c-8329-e11d-7e53-afbec9146156). Runal and Claro are classified as susceptible cultivars. Arina is considered intermediate, whereas Titlis and Camedo are described as resistant cultivars to Z. tritici. On the left panel, ΔZtKu70 and ΔZtSof1 showed comparable host damage for the Runal, Claro, and Titlis cultivars at 14 dpi. Both strains were avirulent in Arina and Camedo. On the right panel, the asexual reproductive structures were observed within the necrosis of those plants inoculated with the ΔZtKu70 strain after 21 days of infection. No pycnidium was observed for plants sprayed with the ΔZtSof1 mutant, demonstrating that the failure to undergo asexual reproduction is associated with the disruption of ZtSof1 per se than a cultivar-specific interaction. [file 12915_2020_838_MOESM11_ESM.jpg]

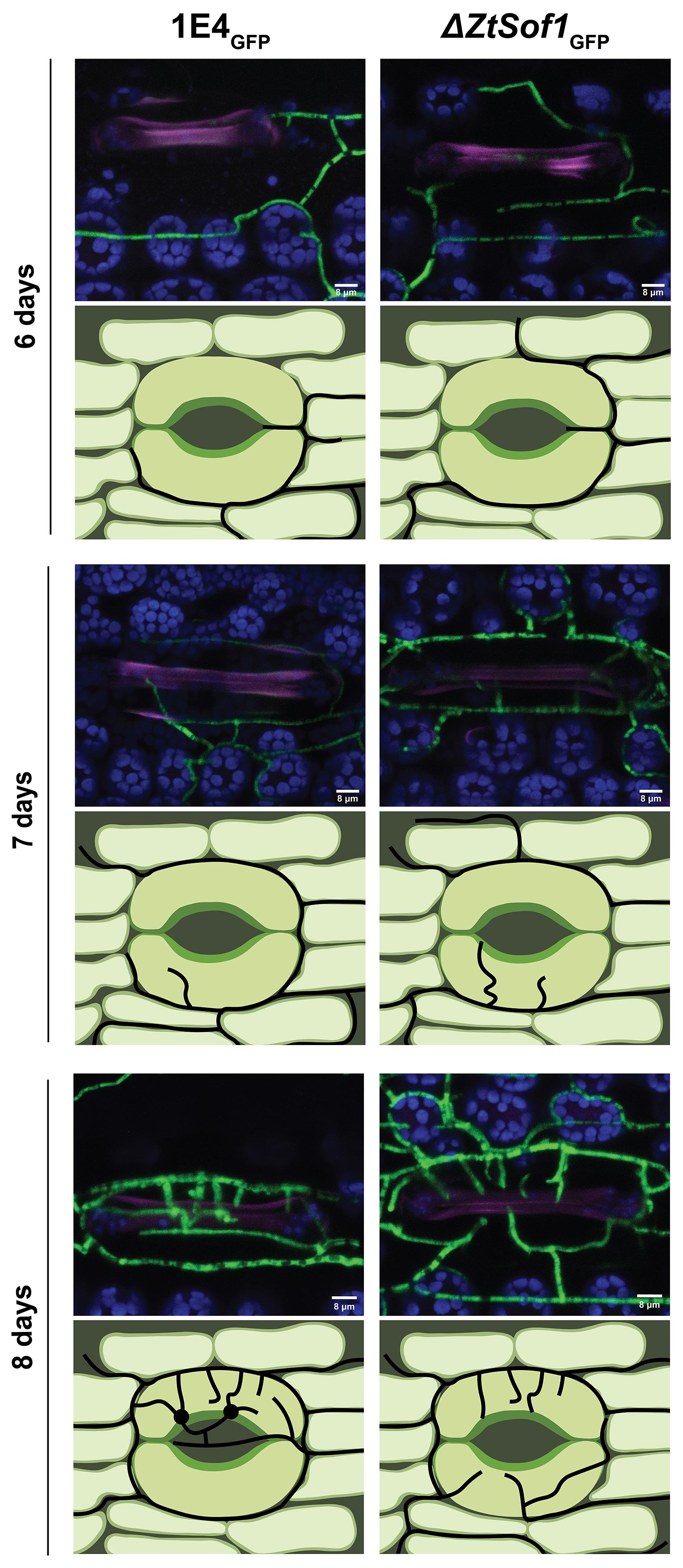

Supplement: Supplementary file 12 — Additional file 12: Figure S12. Confocal microscopy images and schematic demonstration of hyphal penetration, substomatal colonization, and initial stages of pycnidial development. Susceptible wheat cultivar Drifter was inoculated with the fluorescent 1E4GFP (wild-type) and ΔZtSof1GFP strains and monitored by confocal microscopy at different days post-infection (dpi). At 6 dpi, the epiphytic filamentous hyphae penetrate the host tissue through stomatal openings. At 7 dpi, the fungus initiated the intracellular hyphal colonization of the substomatal chamber. The filamentous surrounding the stomatal guard cells produce specialized knots from where secondary hyphae emerge and germinate. Up to this point, none morphological difference of hyphal extension or intracellular hyphal colonization was noticed between 1E4GFP and ΔZtSof1GFP strains. At 8 dpi, the secondary hyphae fuse with another nearby hypha (represented by black circles) in the 1E4GFP strain, creating an interconnected network in the sub-stomatal cavity. Unlike, the secondary hyphae of the ΔZtSof1GFP mutant kept extending as individual filamentous. No anastomosis was observed until this developmental stage. For later time points (9 and 12 dpi), please see Figure 6. [file 12915_2020_838_MOESM12_ESM.jpg]

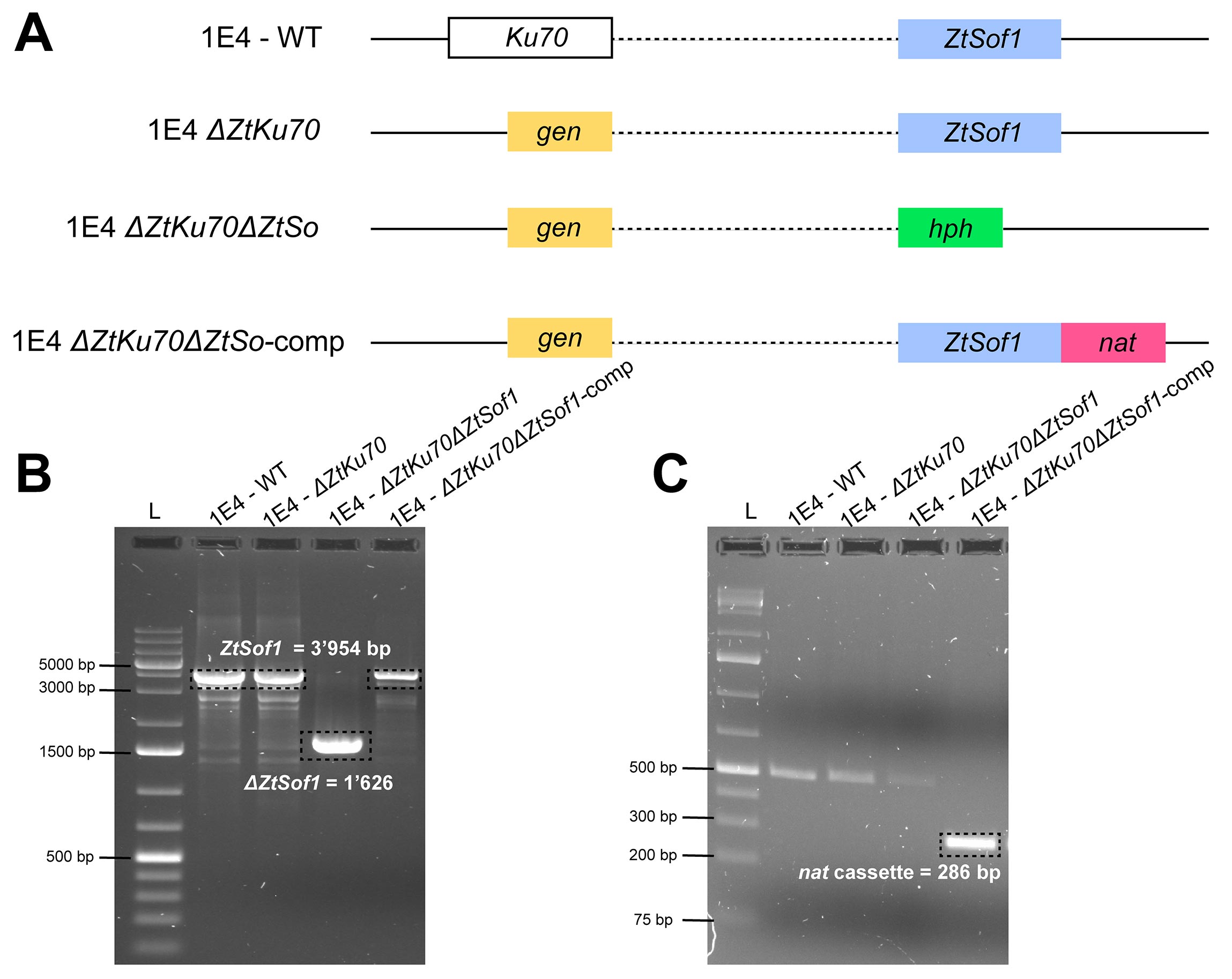

Supplement: Supplementary file 13 — Additional file 13: Figure S13. Description of functional characterizations performed in this study. (A) To increase the homologous recombination efficiency, we first inactivated the ZtKu70 (Mycgr3G85040 or Zt09_3_00215) gene via homologous recombination in the 1E4 wild-type (WT) strain using the plasmid pGEN-YR-ΔZtKu70 [80], containing a geneticin resistance gene cassette (also known as G418), as a selectable marker. To disrupt the Z. tritici So gene (ZtSof1), 1 Kb size of both flanking regions were amplified from the 1E4-WT genomic DNA. The hygromycin resistance gene cassette (hph), used as a selective marker, was amplified from pES6 plasmid (obtained from E. H. Stukenbrock, Kiel University, unpublished). The pES1 plasmid (obtained from E. H. Stukenbrock, Kiel University, unpublished) was digested with KpnI and SbfI for plasmid linearization, and three fragments were assembled, which resulted in the pES1-ΔZtSof1. The ZtSof1 gene was knocked-out via homologous recombination in the genetic background of the 1E4ΔZtKu70 mutant, generating the double mutant 1E4ΔZtKu70ΔZtSof1. To reintroduce the ZtSof1 gene into the ΔZtSof1 mutant strain, we used the plasmid pES1. We amplified the nourseothricin resistance gene cassette (nat) from pES43 plasmid (obtained from E. H. Stukenbrock, Kiel University, unpublished) to be used as a selectable marker. ZtSof1 gene containing 1 Kb size of each flank region and the nat resistance gene cassette were assembled into pES1, resulting in pES1-ΔZtSof1-comp that allowed to introduce the ZtSof1 gene into its native location, generating the 1E4ΔZtKu70ΔZtSof1-comp mutant. (B) Agarose gel shows the PCR fragments at the expected sizes of 3’954 base pairs (bp) or 1’626 bp, confirming the presence of the ZtSof1 native gene or the hygromycin resistance gene, respectively. (C) Agarose gel shows a PCR fragment of 286 bp, confirming the presence of the nourseothricin resistance gene only in the 1E4ΔZtKu70ΔZtSof1-comp mutant. [file 12915_2020_838_MOESM13_ESM.jpg]
